# Population Sepsis Incidence, Mortality, and Trends in Hong Kong Between 2009 and 2018 Using Clinical and Administrative Data

**DOI:** 10.1093/cid/ciad491

**Published:** 2023-08-19

**Authors:** Lowell Ling, Jack Zhenhe Zhang, Lok Ching Chang, Lok Ching Sandra Chiu, Samantha Ho, Pauline Yeung Ng, Manimala Dharmangadan, Chi Ho Lau, Steven Ling, Man Yee Man, Ka Man Fong, Ting Liong, Alwin Wai Tak Yeung, Gary Ka Fai Au, Jacky Ka Hing Chan, Michele Tang, Ying Zhi Liu, William Ka Kei Wu, Wai Tat Wong, Peng Wu, Benjamin J Cowling, Anna Lee, Chanu Rhee

**Affiliations:** Department of Anesthesia and Intensive Care, The Chinese University of Hong Kong, Hong Kong SAR, China; Department of Anesthesia and Intensive Care, The Chinese University of Hong Kong, Hong Kong SAR, China; Department of Anesthesia and Intensive Care, The Chinese University of Hong Kong, Hong Kong SAR, China; Department of Anesthesia and Intensive Care, The Chinese University of Hong Kong, Hong Kong SAR, China; Department of Anesthesia and Intensive Care, The Chinese University of Hong Kong, Hong Kong SAR, China; Critical Care Medicine Unit, The University of Hong Kong, Hong Kong SAR, China; Department of Adult Intensive Care, Queen Mary Hospital, Hong Kong SAR, China; Department of Intensive Care, Princess Margaret Hospital, Hong Kong SAR, China; Department of Intensive Care, North District Hospital, Hong Kong SAR, China; Department of Intensive Care, Tuen Mun Hospital, Hong Kong SAR, China; Department of Intensive Care, Pamela Youde Nethersole Eastern Hospital, Hong Kong SAR, China; Department of Intensive Care, Queen Elizabeth Hospital, Hong Kong SAR, China; Department of Intensive Care, United Christian Hospital, Hong Kong SAR, China; Department of Medicine and Geriatrics, Ruttonjee and Tang Shiu Kin Hospitals, Hong Kong SAR, China; Department of Intensive Care, Kwong Wah Hospital, Hong Kong SAR, China; Department of Medicine, Tseung Kwan O. Hospital, Hong Kong SAR, China; Department of Medicine and Geriatrics, Caritas Medical Centre, Hong Kong SAR, China; Department of Anesthesia and Intensive Care, The Chinese University of Hong Kong, Hong Kong SAR, China; Department of Anesthesia and Intensive Care, The Chinese University of Hong Kong, Hong Kong SAR, China; State Key Laboratory of Digestive Diseases, Li Ka Shing Institute of Health Sciences, The Chinese University of Hong Kong, Hong Kong SAR, China; Institute of Digestive Disease, The Chinese University of Hong Kong, Hong Kong SAR, China; CUHK Shenzhen Research Institute, Shenzhen, China; Peter Hung Pain Research Institute, The Chinese University of Hong Kong, Hong Kong SAR, China; Department of Anesthesia and Intensive Care, The Chinese University of Hong Kong, Hong Kong SAR, China; WHO Collaborating Centre for Infectious Disease Epidemiology and Control, School of Public Health, Li Ka Shing Faculty of Medicine, The University of Hong Kong, Hong Kong SAR, China; Laboratory of Data Discovery for Health Limited, Hong Kong Science and Technology Park, New Territories, Hong Kong SAR, China; WHO Collaborating Centre for Infectious Disease Epidemiology and Control, School of Public Health, Li Ka Shing Faculty of Medicine, The University of Hong Kong, Hong Kong SAR, China; Laboratory of Data Discovery for Health Limited, Hong Kong Science and Technology Park, New Territories, Hong Kong SAR, China; Department of Anesthesia and Intensive Care, The Chinese University of Hong Kong, Hong Kong SAR, China; Department of Population Medicine, Harvard Medical School/Harvard Pilgrim Health Care Institute, Boston, Massachusetts, USA; Division of Infectious Diseases, Department of Medicine, Brigham and Women's Hospital, Boston, Massachusetts, USA

**Keywords:** epidemiology, Sepsis-3, surveillance, clinical coding, infections

## Abstract

**Background:**

Sepsis surveillance using electronic health record (EHR)–based data may provide more accurate epidemiologic estimates than administrative data, but experience with this approach to estimate population-level sepsis burden is lacking.

**Methods:**

This was a retrospective cohort study including all adults admitted to publicly funded hospitals in Hong Kong between 2009 and 2018. Sepsis was defined as clinical evidence of presumed infection (clinical cultures and treatment with antibiotics) and concurrent acute organ dysfunction (≥2-point increase in baseline Sequential Organ Failure Assessment [SOFA] score). Trends in incidence, mortality, and case fatality risk (CFR) were modeled by exponential regression. Performance of the EHR-based definition was compared with 4 administrative definitions using 500 medical record reviews.

**Results:**

Among 13 540 945 hospital episodes during the study period, 484 541 (3.6%) had sepsis by EHR-based criteria with 22.4% CFR. In 2018, age- and sex-adjusted standardized sepsis incidence was 756 per 100 000 (relative change: +2.8%/y [95% CI: 2.0%–3.7%] between 2009 and 2018) and standardized sepsis mortality was 156 per 100 000 (relative change: +1.9%/y; 95% CI: .9%–2.8%). Despite decreasing CFR (relative change: −0.5%/y; 95% CI: −1.0%, −.1%), sepsis accounted for an increasing proportion of all deaths (relative change: +3.9%/y; 95% CI: 2.9%–4.8%). Medical record reviews demonstrated that the EHR-based definition more accurately identified sepsis than administrative definitions (area under the curve [AUC]: .91 vs .52–.55; *P* < .001).

**Conclusions:**

An objective EHR-based surveillance definition demonstrated an increase in population-level standardized sepsis incidence and mortality in Hong Kong between 2009 and 2018 and was much more accurate than administrative definitions. These findings demonstrate the feasibility and advantages of an EHR-based approach for widescale sepsis surveillance.

## Teaser

An electronic health record-based sepsis surveillance definition based on objective clinical markers of infection and organ dysfunction demonstrated increasing population-level standardized sepsis incidence and mortality in Hong Kong between 2009–2018 and was much more accurate than administrative definitions on medical record review.

Sepsis is a leading cause of death. The Global Burden of Disease Study estimated that there were 48.9 million cases of sepsis worldwide, contributing to 11 million deaths in 2017 [1]. This study also suggested that global sepsis incidence has decreased by 37% over the last 3 decades. However, many individual regional and national studies report that sepsis incidence rates have been stable or increasing by various magnitudes over time.

Most sepsis epidemiologic studies have utilized administrative codes from hospital discharge databases or death certificates, but these suffer from poor sensitivity and imperfect specificity [2–4]. Administrative data may also generate misleading sepsis incidence and mortality trends due to improvements in sepsis awareness and clinical documentation as well as financial incentives to code for higher complexity [5, 6].

There is growing interest in electronic health record (EHR)–based sepsis surveillance using clinical markers of infection and concurrent organ dysfunction [4]. Multiple studies have shown that EHR-based methods provide more accurate and objective estimates than administrative methods [7–10]. A clinical surveillance definition has previously been applied to EHR data from over 400 hospitals to generate national estimates of sepsis burden in the United States [7].

Population sepsis estimates using EHR-based clinical data outside of the United States, however, are lacking. Furthermore, no study has applied this method to clinical data covering an entire population. In Hong Kong, septicemia is ranked among the top 10 causes of death based on death certificate data and analyses using hospital discharge codes have estimated an annual sepsis incidence of 296.1 per 100 000 [11, 12]. In this study, we applied a previously validated EHR-based surveillance definition to clinical data from a population health database to estimate the age- and sex-adjusted standardized incidence and mortality of adult sepsis in Hong Kong between 2009 and 2018 and compared its performance with administrative methods [10].

## METHODS

### Study Design

We conducted a retrospective cohort study including all adults (≥18 years) admitted to all 41 publicly funded hospitals in Hong Kong between 1 April 2009 and 31 March 2019. Each calendar year started on 1 April and ended on 31 March in the following year to match our public hospitals' statistical year. Cases were identified from the Clinical Data Analysis and Reporting System (CDARS), a population EHR database that includes comprehensive clinical data on 90% of hospital medical care in Hong Kong [10, 13]. This study was approved by The Joint Chinese University of Hong Kong–New Territories East Cluster Clinical Research Ethics Committee with a waiver of informed consent (2019.214) and New Territories West Cluster Research Ethics Committee (NTWC/REC/21004), Hong Kong East Cluster Research Ethics Committee (HKECREC-2020-128), Institutional Review Board of the University of Hong Kong/Hospital Authority Hong Kong West Cluster (HUW 20-815), Research Ethics Committee (Kowloon Central/Kowloon East) (KC/KE-20-0363/ER-1), and Research Ethics Committee of Kowloon West Cluster (KW/EX-21-020(156-02), with a waiver of informed consent.

### Data Extraction

The CDARS contains clinical records of all inpatients and outpatients treated in Hong Kong public hospitals since 1995, including admission and discharge dates, medication records, operation records, laboratory and microbiological tests, and diagnosis and procedure codes. A unique hospital episode was defined from the date of admission to the date of discharge home (alive) or death. Interhospital transfers were considered continuous hospitalizations. Final discharge destination could not be retrieved in 603 (0.004%) of all hospital episodes (n = 13 540 945); these cases were assumed to have survived the hospital episode.

### Primary EHR Sepsis Surveillance Definition

**Figure 1.**
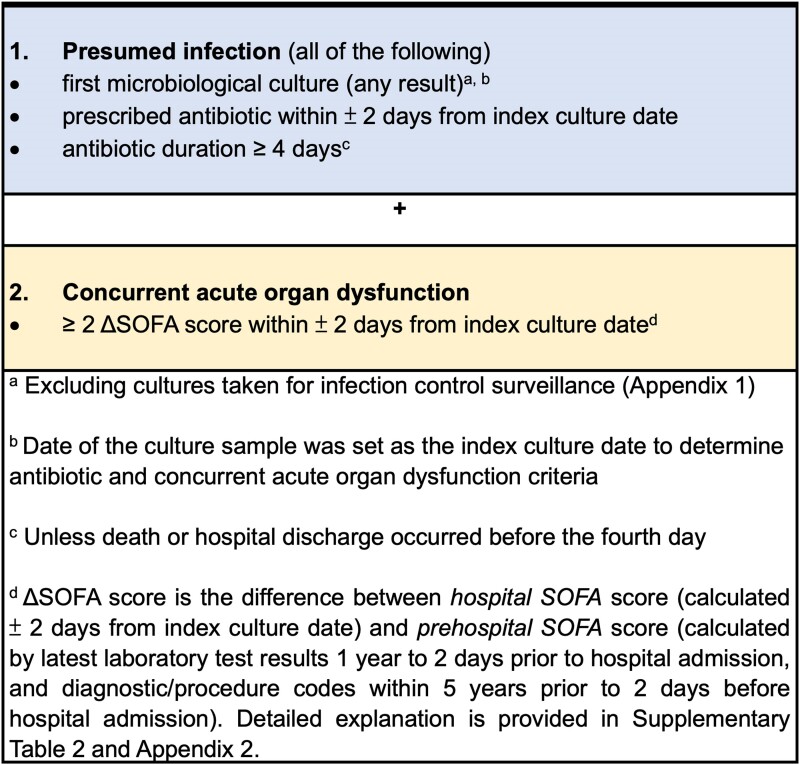
PrimaryEHR sepsis clinical surveillance definition. Abbreviations: EHR, electronic health record; SOFA, Sequential Organ Failure Assessment.

We used our previously validated surveillance definition modeled on the Third International Consensus Definitions of Sepsis and Septic Shock (Sepsis-3) criteria and the US Centers for Disease Control and Prevention (CDC) Adult Sepsis Event definition using the estimated Sequential Organ Failure Assessment (SOFA) score to identify hospital episodes with sepsis (Supplementary Table 1) [10, 14–16]. Sepsis was defined as clinical evidence of (1) presumed infection, defined as any microbiological culture (excluding cultures taken for infection control surveillance purposes [Appendix 1]) and antibiotic started within ±2 calendar days from date of the index culture and continued for at least 4 days (unless death or hospital discharge occurred before the fourth day), and (2) concurrent acute organ dysfunction, defined as a change in (Δ) SOFA score of 2 or more during the infection episode (within ±2 calendar days of the index culture date) from prehospital baseline (Figure 1) [10, 14]. A detailed explanation of the calculation of prehospital and hospital SOFA scores using objective clinical data (laboratory results including bilirubin, creatinine, platelet, Glasgow Coma Scale [GCS] and ratio of arterial oxygen partial pressure to the fraction of inspired oxygen [P_a_O_2_/F_i_O_2_], medication records) and diagnosis/procedure codes is provided in Supplementary Table 2 and Appendix 2. Hospital episodes that had presumed infection but a ΔSOFA score less than 2 were defined as “uncomplicated infection.” The “all infection” group included all cases of “uncomplicated infection” and “sepsis.” Consistent with previous studies, missing laboratory data were assumed to be normal (Supplementary Table 3) [7].

### Other Sepsis Surveillance Methods

We applied an additional 4 administrative methods and 2 secondary variations of the primary EHR-based definition to the dataset to compare differences in estimated sepsis burden. Administrative methods using International Classification of Diseases (ICD) codes included “Implicit” [17], “Explicit” [1, 7, 17], “Martin” [2], and “Local sepsis codes” (Appendixes 3–6). Minor modifications were required because modified International Classification of Diseases, Ninth Revision, Clinical Modification (ICD-9-CM), coding is used in CDARS [18]. Two variations of the primary EHR-based definition included the following: (1) “No prehospital SOFA” (only using a ≥2 hospital SOFA score to identify sepsis) and (2) “Only objective data” (only using clinical data [bilirubin, platelet, creatinine, GCS, P_a_O_2_/F_i_O_2_, vasopressor drug record but no diagnosis/procedure codes] to calculate ΔSOFA score, in order to minimize potential ascertainment bias related to changing coding practices over time) [19].

### Validation Cohort

The primary EHR-based definition was previously validated using a single-center cohort from Hong Kong [10]. To ensure generalizability across all included hospitals, we evaluated the performance of all 7 sepsis surveillance methods with a 500 validation cohort consisting of 50 randomly selected hospital episodes with infection across all hospitals from each study calendar year. Two physicians blinded to the primary EHR method independently reviewed the medical records to determine presence of sepsis (Appendix 7) [14]. Disagreements were resolved by discussion and arbitrated by a third clinician if agreement could not be reached. Performances of all methods were assessed by sensitivity, specificity, positive predictive value (PPV), negative predictive value (NPV), and area under the curve (AUC) using physician consensus as the gold standard. Reasons for misclassification of the primary her method were recorded.

### Statistical Analysis

The Charlson Comorbidity Index (CCI) was calculated using diagnostic codes [20]. Site of infection was defined as either positive microbiological culture test from that site or an infection diagnosis code (Appendix 8). Mortality was defined as all-cause mortality at hospital discharge.

Standardized incidence and mortality were calculated by direct standardization to adjust for age and sex using Hong Kong's year 2008 population structure as reference (Supplementary Table 4) [21]. Exponential regression was used to model the relative annual change of all incidence or mortality estimates. Case fatality risk (CFR) was defined as the number of all-cause deaths at hospital discharge divided by total number of cases in each group. Proportions of sepsis-related deaths were calculated by dividing number of all-cause sepsis-related deaths by all deaths among the Hong Kong population, all hospital episodes, and all infection [21].

Cohen's kappa coefficient was used to measure interobserver agreement between 2 physicians on classification of sepsis. Differences in AUCs were assessed using DeLong's test. Wilcoxon’s rank-sum test was used to compare SOFA scores between groups. Difference in mortality was assessed by chi-square test. For all statistical tests, *P* < .05 (2-sided) was considered statistically significant.

## RESULTS

### Characteristics of the Study Cohort

Between 1 April 2009 and 31 March 2019, a total of 13 540 945 adult hospital episodes comprising 2 928 757 unique patients were identified from CDARS (Supplementary Figure 1). There were 2 373 393 (17.5%) cases of all infection, including 1 888 852 (13.9%) cases of uncomplicated infection and 484 541 (3.6%) cases of sepsis based on the primary EHR-based sepsis definition (Table 1).

**Table 1.** Demographic and Clinical Characteristics of the Study Cohort

	Sepsis (n = 484 541)	Uncomplicated Infection (n = 1 888 852)	All Infection (N = 2 373 393)
Age, median (IQR), y	77 (63–85)	74 (57–84)	75 (58–84)
Age group			
18–29 y	9130 (1.9)	91 808 (4.9)	100 938 (4.3)
30–39 y	13 201 (2.7)	114 815 (6.1)	128 016 (5.4)
40–49 y	22 970 (4.7)	124 011 (6.6)	146 981 (6.2)
50–59 y	49 074 (10.1)	207 270 (11.0)	256 344 (10.8)
60–69 y	74 301 (15.3)	274 892 (14.6)	349 193 (14.7)
70–79 y	106 432 (22.0)	372 922 (19.7)	479 354 (20.2)
≥80 y	209 433 (43.2)	703 134 (37.2)	912 567 (38.4)
Sex			
Male	284 648 (58.7)	948 328 (50.2)	1 232 976 (52.0)
Female	199 893 (41.3)	940 524 (49.8)	1 140 417 (48.1)
CCI, median (IQR)	0 (0–2)	0 (0–2)	0 (0–2)
Comorbidities			
Diabetes mellitus	93 333 (19.3)	354 682 (18.8)	448 015 (18.9)
Cardiovascular disease	147 652 (30.5)	513 475 (27.2)	661 127 (27.9)
Malignancy	70 329 (14.5)	243 849 (12.9)	314 178 (13.2)
Liver disease	43 077 (8.9)	120 121 (6.4)	163 198 (6.9)
Renal disease	37 324 (7.7)	165 983 (8.8)	203 307 (8.6)
Connective tissue disease	4584 (0.9)	22 872 (1.2)	27 456 (1.2)
Chronic pulmonary disease	67 902 (14.0)	310 600 (16.4)	378 502 (15.9)
HIV/AIDS	665 (0.1)	2949 (0.2)	3614 (0.2)
Community-acquired	428 637 (88.5)	1 697 426 (89.9)	2 126 063 (89.6)
Positive microbiological culture	216 096 (44.6)	698 880 (37.0)	914 976 (38.6)
Positive blood culture	56 485 (11.7)	53 409 (2.8)	109 894 (4.6)
Site of infection			
Respiratory	169 680 (35.0)	551 208 (29.2)	720 888 (30.4)
Urinary	128 521 (26.5)	455 964 (24.1)	584 485 (24.6)
Gastrointestinal	61 644 (12.7)	119 423 (6.3)	181 067 (7.6)
Musculoskeletal	11 632 (2.4)	79 538 (4.2)	91 170 (3.8)
Neurological	1774 (0.4)	3216 (0.2)	4990 (0.2)
Cardiac	1126 (0.2)	1238 (0.1)	2364 (0.1)
Eye/dental/ENT	2219 (0.5)	8555 (0.5)	10 774 (0.5)
Prosthesis	689 (0.1)	5476 (0.3)	6165 (0.3)
Skin	11 966 (2.5)	96 307 (5.1)	108 273 (4.6)
Systemic	59 524 (12.3)	62 366 (3.3)	121 890 (5.1)
Unknown	148 708 (30.7)	726 616 (38.5)	875 324 (36.9)
Prehospital SOFA score, median (IQR)	0 (0–1)	0 (0–2)	0 (0–2)
Hospital SOFA score, median (IQR)	3 (2–5)	1 (0–2)	1 (0–3)
Number of organ dysfunctions			
0	0 (0)	939 152 (49.7)	939 152 (39.6)
1	115 680 (23.9)	715 424 (37.9)	831 104 (35.0)
2	209 144 (43.2)	203 673 (10.8)	412 817 (17.4)
3	116 500 (24.0)	28 984 (1.5)	145 484 (6.1)
≥4	43 217 (8.9)	1619 (0.1)	44 836 (1.9)
Vasopressor	44 948 (9.3)	0 (0)	44 948 (1.9)
Mechanical ventilation	69 422 (14.3)	2207 (0.1)	71 629 (3.0)
Renal replacement therapy	17 366 (3.6)	43 181 (2.3)	60 547 (2.6)
ICU admission	62 713 (12.9)	18 740 (1.0)	81 453 (3.4)
28-Day mortality	95 892 (19.8)	109 184 (5.8)	205 076 (8.6)
Hospital episode mortality	108 768 (22.4)	109 788 (5.8)	218 556 (9.2)
Hospital episode length of stay, median (IQR), d	10 (5–21)	5 (2–11)	5 (3–13)

Comorbidities and the CCI of each hospital episode were calculated using all diagnosis and procedure codes within 5 y prior to 2 d before the hospital admission from the Clinical Data Analysis and Reporting System. All values are expressed as n (%) unless otherwise specified.

Abbreviations: CCI, Charlson Comorbidity Index; ENT, eye, nose, and throat; HIV, human immunodeficiency virus; ICU, intensive care unit; IQR; interquartile range; SOFA, Sequential Organ Failure Assessment.

^a^Defined as index culture date ≤3 d after hospital episode admission date.

^b^Any positive microbiological culture result within ±2 d of index culture date. Coagulase-negative *Staphylococcus aureus* was only counted as a true pathogen in blood cultures if grown ≥2 times from blood cultures within 7 d of the first sample.

^c^Any positive blood culture result within ±2 d of index culture date. The rates of positive blood cultures among sepsis cases and uncomplicated infection cases that had blood cultures performed were 18.2% (56 485/310 955) and 6.5% (53 409/816 590), respectively.

^d^Compared with uncomplicated infection cases, sepsis cases had higher median [IQR] hospital SOFA scores (3 [2–5] vs 1 [0–2]; *P* < .001).

^e^Calculated based on the number of hospital component SOFA scores ≥1.

^f^Defined as all-cause death on or before 28 d after index culture date.

Overall, 54.9% (54 701/99 632) of sepsis cases that required either mechanical ventilation or vasopressors were not managed in the intensive care unit (ICU). Among sepsis cases treated with mechanical ventilation or vasopressors, those managed in general wards had higher mortality than those admitted to ICUs (54.3% [29 729/54 701] vs 30.0% [13 481/44 931]; *P* < .001). Most sepsis cases (43.2%; 209 144/484 541) had 2 organ dysfunctions during the hospital episode with an 18.4% CFR (38 381/209 144), while 8.9% (43 217/484 541) of sepsis cases who had 4 or more organ dysfunctions had the highest CFR at 47.3% (20 428/43 217) (Supplementary Figure 2).

### Incidence, Mortality, and Trends of Sepsis

**Figure 2.**
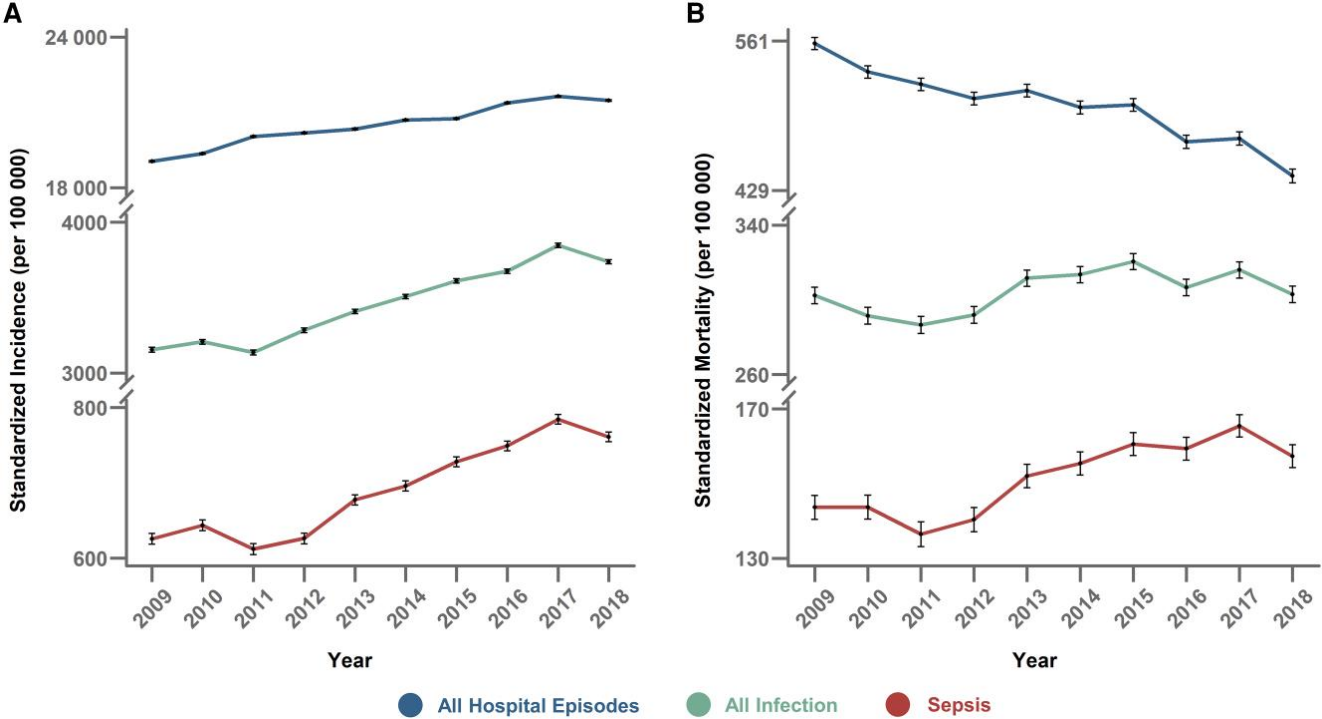
Trends in annual age- and sex-adjusted standardized incidence (*A*) and mortality (*B*) of sepsis; all infection and all hospital episodes are expressed as per 100 000 of the Hong Kong population using the year 2008 population structure as reference. Error bars are shown as 95% CIs of the point estimate. Abbreviaton: CIs, confidence intervals.

Standardized sepsis incidence and mortality trends estimated by the primary EHR method are shown in Figure 2 and Supplementary Table 5. Sepsis incidence increased from 623 per 100 000 in 2009 to 756 per 100 000 in 2018 (relative change: +2.8%/y; 95% CI: 2.0%–3.7%; *P* < .001). The proportion of all hospital episodes with sepsis increased from 3.3% to 3.9% (relative change: +2.4%/y; 95% CI: 1.4%–3.3%; *P* < .001) (Supplementary Table 6 and Supplementary Figure 3). Sepsis mortality increased from 142 per 100 000 in 2009 to 156 per 100 000 in 2018 (relative change: +1.9%/y; 95% CI: .9%–2.8%; *P* = .002).

**Figure 3.**
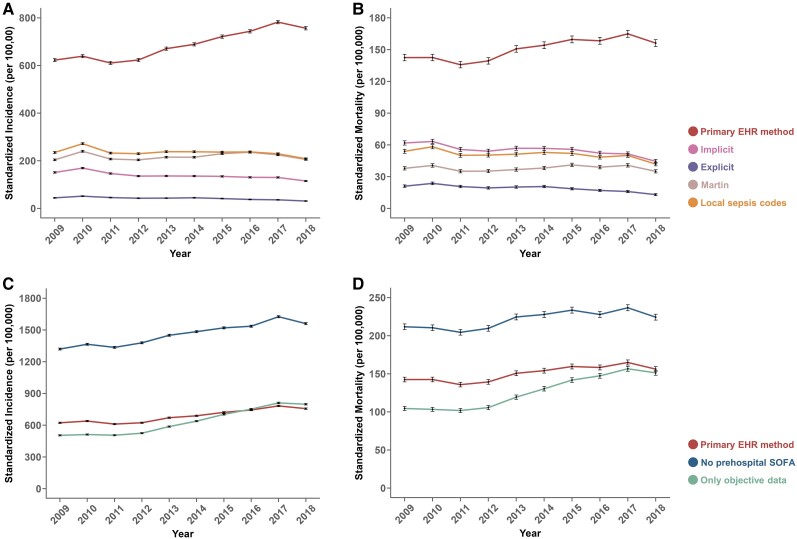
Trendsin annual age- and sex-adjusted standardized incidence and mortality estimates are expressed as per 100 000 of the Hong Kong population using the year 2008 population structure as reference. Standardized incidence (*A*) and mortality (*B*) of sepsis estimated by 4 administrative methods were compared with the primary EHR-based sepsis surveillance method. Standardized incidence (*C*) and mortality (*D*) estimated by 2 secondary variations of the primary EHR-based method were compared with the primary EHR sepsis surveillance method. Error bars are shown as 95% CIs of the point estimate. Abbreviations: CIs, confidence intervals; EHR, electronic health record; SOFA, Sequential Organ Failure Assessment.

Sepsis trends estimated by different methods are shown in Figure 3 and Supplementary Table 7. Both “Implicit” (relative change: −2.9%/y; 95% CI: −4.2%, −1.6%; *P* < .001) and “Explicit” (relative change: −4.0%/y; 95% CI: −5.8%, −2.2%; *P* = .001) methods showed a decrease in sepsis incidence. Similarly, both “Implicit” (relative change: −2.8%/y; 95% CI: −4.1%, −1.4%; *P* = .001) and “Explicit” (relative change: −4.8%/y; 95% CI: −6.9%, −2.7%; *P* < .001) methods showed a decrease in sepsis mortality. Sepsis incidence and mortality estimates were much higher for the secondary EHR-based definition “No prehospital SOFA.”

**Figure 4.**
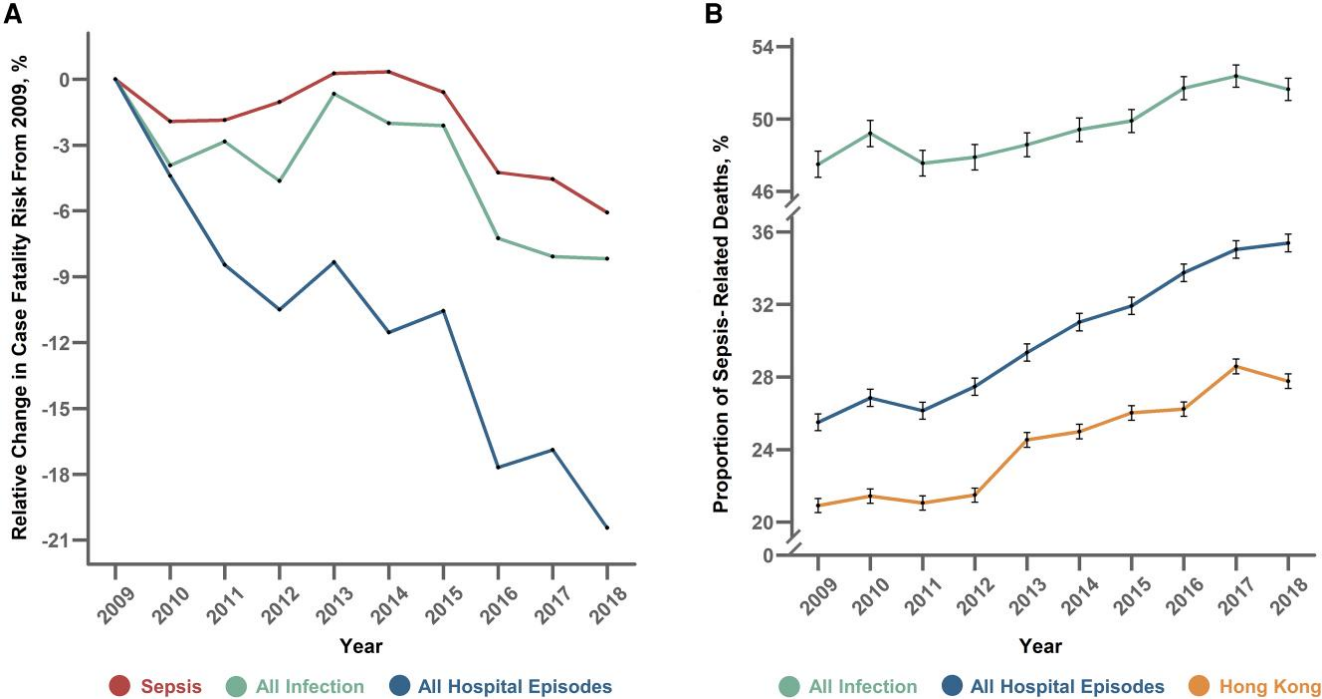
*A*, Relative change in case fatality risk from year 2009 of sepsis (identified by primary EHR-based definition), all infection, and all hospital episodes at hospital episode discharge. *B*, Annual proportion of deaths due to sepsis among all infection deaths, all hospital episode deaths, and Hong Kong deaths. Error bars are shown as 95% CIs of the point estimate. Abbreviations: CIs, confidence intervals; EHR, electronic health record.

Sepsis CFR slightly declined from 23.0% to 21.6% (relative change: −0.5%/y; 95% CI: −1.0%, −.1%; *P* = .03) between 2009 and 2018 (Figure 4, Supplementary Table 8 and Supplementary Figure 4). During this period, there was a greater reduction in CFR among all hospital episodes from 3.0% to 2.4% (relative change: −2.2%/y; 95% CI: −2.7%, −1.6%; *P* < .001). Overall, the proportion of sepsis-related deaths increased among all hospital episode deaths (relative change: +4.1%/y; 95% CI: 3.5%–4.7%; *P* < .001) and Hong Kong deaths (relative change: +3.9%/y; 95% CI: 2.9%–4.8%; *P* < .001).

### Surveillance Performance in the Validation Cohort

Two independent physicians had good initial interrater agreement (*k* = .83; 95% CI: .77–.89) on sepsis classification. Consensus was reached after discussion between the 2 reviewers for all cases. While specificities of the primary EHR-based and administrative methods were all 0.96 or greater, the sensitivity of administrative methods was only 5%–15% compared to 84% of the primary EHR-based definition (Table 2). In addition, the primary EHR method had the highest AUC of .91 (95% CI: .87– .95) to distinguish sepsis among all infection cases compared with other methods (*P* < .001). The primary EHR method misclassified 4.4% (22/500) of cases, most commonly due to inability to identify oxygen therapy (Supplementary Table 9). The “Only objective data” definition had similar performance (AUC: .89; 95% CI: .84–.93; *P* = .14) to the primary EHR method.

**Table 2.** Performance of Different Sepsis Surveillance Methods Using a Validation Cohort

	Primary EHR Method	Implicit	Explicit	Martin	Local Sepsis Codes	No Prehospital SOFA	Only Objective Data
Sensitivity	.84 (95% CI: .76, .91)	.12 (95% CI: .06, .19)	.05 (95% CI: .01, .10)	.12 (95% CI: .06, .19)	.15 (95% CI: .08, .22)	.95 (95% CI: .91, .99)	.79 (95% CI: .70, .87)
Specificity	.99 (95% CI: .97, 1.00)	.99 (95% CI: .97, 1.00)	.99 (95% CI: .98, 1.00)	.96 (95% CI: .94, .98)	.96 (95% CI: .94, .98)	.73 (95% CI: .69, .78)	.99 (95% CI: .97, 1.00)
PPV	.93 (95% CI: .88, .98)	.67 (95% CI: .45, .88)	.56 (95% CI: .23, .88)	.41 (95% CI: .24, .59)	.46 (95% CI: .29, .62)	.47 (95% CI: .40, .53)	.93 (95% CI: .87, .98)
NPV	.96 (95% CI: .94, .98)	.82 (95% CI: .79, .86)	.81 (95% CI: .78, .85)	.82 (95% CI: .78, .85)	.82 (95% CI: .79, .86)	.98 (95% CI: .97, 1.00)	.95 (95% CI: .93, .97)
PLR	56.06 (95% CI: 25.22, 124.64)	8.20 (95% CI: 3.16, 21.32)	5.13 (95% CI: 1.40, 18.74)	2.90 (95% CI: 1.43, 5.86)	3.42 (95% CI: 1.79, 6.54)	3.57 (95% CI: 3.01, 4.22)	52.64 (95% CI: 23.63, 117.26)
NLR	.17 (95% CI: .11, .26)	.89 (95% CI: .83, .96)	.96 (95% CI: .92, 1.01)	.92 (95% CI: .85, .99)	.89 (95% CI: .81, .97)	.07 (95% CI: .03, .16)	.22 (95% CI: .15, .32)
AUC	.91 (95% CI: .87, .95)	.55 (95% CI: .52, .59)	.52 (95% CI: .50, .54)	.54 (95% CI: .51, .57)	.55 (95% CI: .52, .59)	.84 (95% CI: .81, .87)	.89 (95% CI: .84, .93)

The validation cohort (n = 500) consisted of 50 randomly selected hospital episodes with infection from each calendar year between 2009 and 2018. Two physicians were blinded to the primary EHR-based method, and independently reviewed the medical records including clinical notes, laboratory results, imaging, and medication orders to determine whether the patient had sepsis (Sepsis-3 criteria). Presence of infection was based on clinical diagnosis with or without confirmatory microbiology. Physicians calculated SOFA scores within ±2 calendar days of the index culture date to determine presence of sepsis. Compared with all administrative methods (implicit, explicit, Martin, local sepsis codes), our primary EHR method had significantly higher AUC (Delong's test, *P* < .001). “Only objective data,” which is a variation of the primary EHR method without use of administrative data, had similar sepsis surveillance performance compared with the primary EHR-based definition (AUC: .89; 95% CI: .84, .93; *P* = .14). “No prehospital SOFA,” which is another variation of the primary EHR method without prehospital SOFA score, had a lower AUC (AUC: .84; 95% CI: .81, .87; *P* = < .001).

Abbreviations: AUC, area under curve; EHR, electronic health record; NLR, negative likelihood ratio; NPV, negative predictive value; PLR, positive likelihood ratio; PPV, positive predictive value; Sepsis-3, Third International Consensus Definitions of Sepsis and Septic Shock; SOFA, Sequential Organ Failure Assessment.

## DISCUSSION

In this 10-year retrospective cohort study of 13 540 945 adult hospital episodes, an EHR-based sepsis surveillance method showed that standardized population sepsis incidence increased exponentially by 2.8% per year and mortality increased exponentially by 1.9% per year in Hong Kong. Despite a relative decline of 0.5% per year in sepsis CFR, the proportion of sepsis-related deaths among all deaths in Hong Kong increased by 3.9% per year. Medical record reviews confirmed that the EHR-based definition more accurately identified sepsis compared with administrative methods.

This is the first study to apply an EHR-based sepsis definition to comprehensive clinical data to estimate sepsis burden in an entire population. Using this method, we found that Hong Kong's standardized sepsis incidence was 756 per 100 000 in 2018, which is in line with the estimated global annual sepsis incidence of 677.5 per 100 000 [1]. This rate is also similar to rates reported from Sweden (780 per 100 000) and Taiwan (772), but higher than France (403), China (422), Spain (445), England (102), New Zealand (107), Norway (140), Brazil (290), and South Korea (453), and much lower than Australia (1163) and Malawi (1772) [22–33].

Apart from intrinsic differences in population health, healthcare resources, and infection epidemiology, major differences in surveillance methodology may account for variations in reported sepsis incidences across different regions [1, 34]. First, study sample coverages have been variable. A German study showed a 10-fold variation in sepsis burden between different districts of the same country [35]. Therefore, studies based on complete regional and population data may be more representative of actual incidence [23, 30–33, 36]. Meanwhile, representative cohorts from national databases may be affected by sampling bias [24, 26]. Second, study settings have been diverse, with some studies confined to the ICU setting (England and Brazil) or the emergency department (Malawi) [22, 26, 28]. It is difficult to derive overall sepsis burden from these settings alone as they represent where patients at extreme ends of sepsis severity are treated.

Our results highlight the potential limitations of utilizing administrative data to estimate population-level sepsis burden. Administrative methods significantly underestimated sepsis incidence in our healthcare setting, with a low sensitivity of 15%. In contrast, the primary EHR method provided reliable sepsis identification with 84% sensitivity and 99% specificity. Correspondingly, our EHR-based sepsis incidence estimates were generally higher than studies from France, Spain, South Korea, Brazil, Norway, and New Zealand, which relied on administrative methods alone [23–25, 27–29]. Instead, we found comparable sepsis incidence to estimates based on objective clinical data in Sweden and Beijing [32, 37]. More specifically, both “Implicit” and “Explicit” methods underestimated sepsis burden in Hong Kong. In contrast, in the United States, implicit codes overestimate while explicit codes underestimate sepsis burden when compared with surveillance using objective clinical data [7]. The same divergence between implicit and explicit methods has been demonstrated in South Korea [24, 38].

Bias in administrative coding due to changes in coding practice and sepsis awareness over time has contributed to exaggerated increases in reported sepsis incidence and declines in CFR compared with objective clinical data in the United States [8, 19, 39]. Interestingly, we observed the opposite trend, such that sepsis incidence using administrative definitions appeared to decrease over time while EHR sepsis surveillance showed that annual incidence has increased. This corresponds to the very low sensitivity of administrative definitions that we found and may reflect factors specific to Hong Kong, including the lack of dedicated coding teams, inadequate training on diagnostic coding, and independence from public healthcare funding. Similar discrepancies between objective prescription of antihypertensive drugs and diagnostic coding for hypertension have been found in Hong Kong's population health database [40]. Our results reinforce the importance of assessing the reliability of different sepsis surveillance methods in individual regions and countries and the potential for confounding when comparing sepsis rates based on administrative definitions in places where coding practices may differ substantially.

A strength of the primary EHR method is the ability to capture pre-existing organ dysfunction prior to hospitalization. As expected, omission of prehospital organ dysfunction reduced specificity and overestimated sepsis burden. Furthermore, administrative methods are often unable to quantify changes in organ functions from codes alone—for example, worsening thrombocytopenia during hospitalization from pre-existing thrombocytopenia. Sepsis surveillance methods should ideally incorporate prehospital data to minimize this systematic bias.

Estimates from different regions and countries consistently showed that sepsis accounted for just 1%–6% of hospitalizations [7, 25, 27, 41–44]. Similarly, sepsis contributed to a relatively minor 3.9% of adult hospitalizations in Hong Kong. Yet, sepsis-related deaths accounted for a disproportionately large proportion (35.4%) of all hospital deaths in 2018. This is consistent with US estimates from both EHR-based definitions and detailed medical record reviews [7, 45]. On a population level, 27.8% of all deaths in Hong Kong were fully or partly attributable to sepsis in 2018, which is 20% higher than official figures gathered from death certificates (combination of pneumonia or septicemia) [12]. Worryingly, death from sepsis was an increasing cause of death despite a slight improvement in sepsis CFR (relative change: −0.5%/y). This is because the reduction in sepsis CFR was almost 5 times lower than the decline in CFR of all adult hospitalizations. Although the decreasing trend of sepsis CFR was consistent with that of France, Australia, Taiwan, and Japan, the magnitude of reduction was 4–10 times lower in Hong Kong [29–31, 41]. Since these are all high-income settings, large variances in estimated CFR reduction may be partly due to differences in approach to sepsis surveillance.

Timely admission to the ICU may help improve sepsis survival [46]. Hong Kong has a significant healthcare resource deficit to treat sepsis, as 54.9% of sepsis cases that required vasopressors or mechanical ventilation were managed on general wards rather than ICUs. Overall, only 12.9% of sepsis cases are managed in the ICU, which is much lower than in the United States, at 54.7%, but comparable to Japan (17.1%) and Beijing (13.8%) [7, 37, 41]. This may be because Hong Kong, Japan, and China have far fewer ICU beds than the United States [47, 48]. Critically ill patients are often triaged from ICU care in Hong Kong due to resource limitations [49, 50]. Our results highlight the need to increase the provision of critical care in Hong Kong to match sepsis burden.

This study has several limitations. First, only public hospital admissions were included. However, the public sector provides 90% of hospital services in Hong Kong [13]. Second, we used the first microbiological test as the only reference point to determine the presence of sepsis. This reduced our ability to detect hospital-acquired sepsis and likely underestimated the true sepsis incidence. Third, rates of missing data for P_a_O_2_/F_i_O_2_ values were very high since arterial blood gases are not routinely drawn in most patients. The GCS and other vital signs were mostly unavailable in our dataset, forcing us to rely on other proxies for cardiovascular and neurologic dysfunction [7–10]. Future EHR-based sepsis surveillance methods may be able to utilize the ratio of peripheral arterial oxygen saturation (S_p_O_2_) to F_i_O_2_ to calculate respiratory SOFA scores and incorporate GCS and other vital signs, which are increasingly recorded electronically. Fourth, we were unable to capture admission data to 2 publicly funded surgical ICUs. Fifth, the burden of pediatric sepsis was not captured. Sixth, patients with sepsis discharged from Accident and Emergency directly without hospitalization were not included. Seventh, nonbacterial sepsis cases may have been excluded if they did not fulfill the antibiotic criteria. Eighth, the validation cohort was sampled from all infection cases rather than all hospital episodes. This may have caused us to overestimate the true sensitivity of the primary EHR definition. Ninth, our primary EHR method still required the use of some administrative data for SOFA calculation. Nevertheless, the performance of the secondary EHR-based definition using only objective clinical data (laboratory results and vasopressor drug records alone) was comparable to the primary EHR method (AUC: .89 vs .91).

### Conclusions

An objective EHR-based surveillance definition demonstrated an increase in population-level sepsis incidence and mortality in Hong Kong between 2009 and 2018 and was much more accurate than administrative definitions based on medical record reviews. These findings underscore the high burden of sepsis in this region and demonstrate the feasibility and advantages of an EHR-based approach for widescale sepsis surveillance.

## Supplementary Data

Supplementary materials are available at *Clinical Infectious Diseases* online. Consisting of data provided by the authors to benefit the reader, the posted materials are not copyedited and are the sole responsibility of the authors, so questions or comments should be addressed to the corresponding author.

## Supplementary Material

ciad491_Supplementary_Data

## Data Availability

The data that support the findings of this study are available from the corresponding author upon reasonable request and approval from ethics committees.

## Notes

***Author contributions.*** L. L., A. L., and C. R. designed the study and funding acquisition. L. L., P. Y. N., M. D., C. H. L., S. L., M. Y. M., K. M. F., T. L., A. W. T. Y., G. K. F. A., J. K. H. C., and M. T. recruited subjects. L. L., J. Z. Z., L. C. C., and L. C. S. C. collected and analyzed the data. L. L. and S. H. performed the physician validation cohort. L. L., J. Z. Z., P. W., B. J. C., A. L., and C. R. interpreted the data. L. L., J. Z. Z., A. L., and C. R. wrote the first draft of the manuscript. All authors commented on and approved the final manuscript.

***Financial support***. This work was supported by a grant from the Health and Medical Research Fund of the Health Bureau of Hong Kong SAR Government (grant no. 18190381).
