# Supplementary material for: Population Sepsis Incidence, Mortality, and Trends in Hong Kong Between 2009 and 2018 Using Clinical and Administrative Data
Source: Clin Infect Dis. 2023 Aug 19;80(1):91–100. doi: 10.1093/cid/ciad491 (PMC11797015; doi:10.1093/cid/ciad491)
Supplement: ciad491_Supplementary_Data [file ciad491_supplementary_data.docx]

**Supplementary Table 1. Comparisons between Primary EHR Method, Sepsis-3 derivation cohort, and U.S. CDC’s Adult Sepsis Event (ASE) definitions**

|  | **Suspected Infection** | **Organ Dysfunction** |
| --- | --- | --- |
| **Sepsis-3^a^** | - Combination of antibiotics (oral or parenteral) and body fluid culture - if antibiotic ordered first, culture sampling must be taken within 24 hours of antibiotic - if culture sampling first, then antibiotic must be ordered within 72 hours of sampling - Specific time/date of suspected infection was defined as the earliest of culture sampling or antibiotic order | - ≥2 ΔSOFA score from up to 48 hours before to 24 hours after onset of suspected infection |
| **Adult Sepsis Event (ASE)^b^** | - Combination of antibiotics and blood culture - ≥4 qualifying antimicrobial days (QADs) starting within ± 2 calendar days of the date of blood culture - at least one QAD within the ± 2 days window must include a parenteral antibiotic - fewer than 4 QADs allowed if antibiotics continued up through the day of or day prior to death, discharge to another hospital, discharge to hospice, or transfer to another acute care hospital | - ≥1 of the following “eSOFA” within ± 2 calendar days of the date of blood culture: - new vasopressor infusion (specific vasopressor not administered in the prior calendar day) - initiation of invasive mechanical ventilation (> 1 calendar day between mechanical ventilation episodes) - doubling of serum creatinine OR decrease by ≥50% of estimated glomerular filtration rate relative to baseline (excluding patients with end-stage renal disease) - total bilirubin ≥ 33 μmol/L and increase by 100% from baseline - platelet count < 100 x 10^3^ cells/μL and ≥ 50% decline from baseline |
| **Primary EHR Method** | - Combination of antibiotics (oral or parenteral) and microbiological culture (excluding cultures for infection control surveillance) - Specific date of suspected infection was defined by using date of first microbiological culture (index culture date) - antibiotics must be started within ± 2 calendar days of index culture date and continued for ≥ 4 days unless death or hospital discharge occurred before the fourth day | - ≥2 ΔSOFA score within ± 2 calendar days before and after date of suspected infection |
| **Key Differences** | - Primary EHR method used any culture similar to Sepsis-3 (instead of only blood cultures used by ASE) to increase sensitivity - Primary EHR method specified a minimum antibiotic duration of 4 days similar to ASE (instead of one antibiotic dose used by Sepsis-3) to increase specificity for infection - Primary EHR method used window of 2 calendar days before and after index culture date similar to ASE (instead of 1 day before and 3 days after culture date used by Sepsis-3) - Primary EHR method defined suspected infection onset time by first culture date whereas Sepsis-3 used the earliest antibiotic or culture time, and ASE by any of the blood culture dates fulfilling the suspected infection criteria | - Primary EHR method used SOFA score as described in Sepsis-3 rather than eSOFA which is used in ASE - Primary EHR method and ASE both assessed organ dysfunction within time window of ± 2 calendar days before and after date of suspected infection, whereas Sepsis-3 used 48 hours before to 24 hours after onset of suspected infection |

^a^ Sepsis 3 criteria derivation cohort[15]

^b^ Adult Sepsis Event as defined in Hospital Toolkit for Adult Sepsis Surveillance[16]

ASE, Adult Sepsis Event; EHR, electronic health record; SOFA, sequential organ failure assessment.

**Supplementary Table 2. Calculation of ΔSOFA score, prehospital and hospital SOFA score by Primary EHR Method**

| **Prehospital (Baseline) SOFA score** | | |
| --- | --- | --- |
|  | **Score** | **Conditions** |
| **Respiratory**  (codes obtained within 5 years to 2 days before admission) | 0 | Assumed to be zero at baseline |
|  | 2 | Diagnostic code of Supplemental oxygen dependence (V46.1:1) or Dependence on respirator (V46.1:0)  or  Procedure code of Long term oxygen therapy (93.96:2) |
|  | | |
| **Central Nervous System**  (codes obtained within 5 years to 2 days before admission) | 0 | Assumed to be zero at baseline |
|  | 2 | Diagnostic code of dementia-associated codes  (full list in Appendix 2) |
|  | | |
| **Cardiovascular** | 0 | Assumed to be zero at baseline |
|  | | |
| **Liver**  (latest bilirubin (μmol/L) within 1 year to 2 days before admission) | 0 | <20 |
|  | 1 | 20−32 |
|  | 2 | 33−101 |
|  | 3 | 102−204 |
|  | 4 | >204 |
|  | | |
| **Coagulation**  (latest platelet count (x 10^3^/µL) within 1 year to 2 days before admission) | 0 | ≥150 |
|  | 1 | 100−149 |
|  | 2 | 50−99 |
|  | 3 | 20−49 |
|  | 4 | <20 |
|  |  |  |
| **Renal**  (latest creatinine (μmol/L) within 1 year to 2 days before admission or codes obtained within 5 years to 2 days before admission) | 0 | ≤109 |
|  | 1 | 110−170 |
|  | 2 | 171−299 |
|  | 3 | 300−440 |
|  | 4 | >440  or  Procedure code of renal dialysis-associated codes  (full list in Appendix 2) |
|  | | |
| **Hospital SOFA score^a^** | | |
|  | **Score** | **Conditions** |
| **Respiratory**  (lowest P_a_O_2_/F_i_O_2_ (mmHg), if not available then diagnostic/procedural code) | 0 | ≥400  or  Lack of verified P_a_O_2_/F_i_O_2_ |
|  | 1 | 300−399  or  Diagnostic code of Respiratory failure (518.81:0), Type II respiratory failure (518.81:2), Type I respiratory failure (518.81:1), Acute respiratory failure (518.81:4), Supplemental oxygen dependence (V46.1:1), Respiratory acidosis (276.2:3), Respiratory insufficiency (518.82:2), Respiratory failure, postpartum (648.94:870), Respiratory failure, antepartum (648.93:870) |
|  | 2 | 200−299  or  Procedure code of BiPAP (93.90:1), NIPPV (93.91:1), Continuous positive airway pressure (93.90:0), Non-invasive mechanical ventilation (93.99:2), Endotracheal intubation (96.04:0), Respiratory tract intubation (96.05:0), Continuous invasive mech vent-<96 hours (96.71:0)  Invasive mechanical ventilation (96.70:0), Continuous invasive mech vent->96 hours (96.72:0), CPAP titration (89.38:1), BiPAP titration (89.38:2), Respiratory tract intubation (96.05:0), Intermittent positive pressure ventilation (93.91:2), Continuous negative pressure ventilation (93.99:1), High frequ oscillatory ventilation (96.70:1) |
|  | 3 | 100−199 |
|  | 4 | < 100  or  Diagnostic code of Respiratory arrest (799.1:0) |
|  | | |
| **Central Nervous System** | 0 | GCS 15 |
|  | 1 | GCS 13−14  or  Diagnostic code of Delirium (780.09:3), Alteration of consciousness (780.09:0), Confusion (298.9:3), Encephalopathy (348.3:0), Acute delirium (293.0:0), Subacute delirium, multi etiology (293.1:2), Delirium superimposed on dementia (290.11:3), Senile dementia w delirium (290.3:0), Vascular dementia w delirium (290.41:1), Subacute delirium (293.1:0) |
|  | 2 | GCS 10−12  or  Diagnostic code of dementia-associated codes  (full list in Appendix 2) |
|  | 3 | GCS 6−9 |
|  | 4 | GCS <6 |
|  | | |
| **Cardiovascular** | 0 | All patients assumed to be zero unless there is diagnostic coding |
|  | 1 | Diagnostic code of Hypotension (458.9:0) |
|  | 2 | Use of dopamine, dobutamine, norepinephrine, epinephrine, vasopressin, isoprenaline, levosimendan |
|  | | |
| **Liver**  (highest bilirubin (μmol/L)) | 0 | <20 |
|  | 1 | 20−32 |
|  | 2 | 33−101 |
|  | 3 | 102−204 |
|  | 4 | >204 |
|  | | |
| **Coagulation**  (lowest platelet (x 10^3^/µL)) | 0 | ≥150 |
|  | 1 | 100−149 |
|  | 2 | 50−99 |
|  | 3 | 20−49 |
|  | 4 | <20 |
|  | | |
| **Renal**  (highest creatinine (μmol/L)) | 0 | ≤109 |
|  | 1 | 110−170 |
|  | 2 | 171−299 |
|  | 3 | 300−440 |
|  | 4 | >440  or  Procedure code of renal dialysis-associated codes  (full list in Appendix 2) |
|  | | |
| **ΔSOFA Score** | | |
|  | **ΔSOFA Calculation** | |
| **Cardiovascular, Liver,**  **Coagulation** | ΔSOFA Score = Hospital SOFA Score – Prehospital SOFA Score | |
| **Respiratory** | ΔSOFA Score = 0 if any prehospital diagnostic code of Supplemental oxygen dependence (V46.1:1) or Dependence on respirator (V46.1:0) or Procedure code of Long term oxygen therapy (93.96:2)  If not, then ΔSOFA Score = Hospital SOFA Score | |
| **Renal** | ΔSOFA Score = 0 if any prehospital procedure code associated with renal replacement therapy (full list in Appendix 2)  If not, then ΔSOFA Score = Hospital SOFA Score – Prehospital SOFA Score | |
| **Central Nervous System** | ΔSOFA Score = Score based on GCS alone if GCS is available but there is no prehospital or hospital diagnostic code of dementia-associated code (full list in Appendix 2).  ΔSOFA Score = 1 if GCS is available and there is diagnostic code of delirium during hospital episode with any prehospital or hospital diagnostic code of dementia-associated code (full list in Appendix 2)  ΔSOFA Score = 0 if GCS is available without a diagnostic code of delirium during hospital episode but there is either prehospital or hospital diagnostic code of dementia-associated code (full list in Appendix 2)  ΔSOFA Score = 1 if GCS is not available but there is diagnostic code of delirium during hospital episode  ΔSOFA Score = 0 if GCS is not available and there is no diagnostic code of delirium during hospital episode | |

^a^ Calculated based on laboratory values and use of medications within ± 2 days of index culture date. All diagnostic and procedural codes at hospital episode discharge were used. GCS on admission was used for SOFA calculation. GCS, Glasgow coma scale; SOFA, sequential organ failure assessment.

**Supplementary Table 3. Missing data of all infection cohort (n = 2,373,393)**

|  | **Missing, n (%)** |
| --- | --- |
| **Prehospital** |  |
| Bilirubin | 553,969 (23.3) |
| Creatinine | 474,296 (20.0) |
| Platelet | 544,605 (22.9) |
| **Hospital** |  |
| Bilirubin | 167,478 (7.1) |
| Creatinine | 119,547 (5.0) |
| Platelet | 99,126 (4.2) |
| GCS | 1,810,783 (76.3) |
| P_a_O_2_/F_i_O_2_ | 2,342,715 (98.7) |

Missing data was calculated for all infection cases (n = 2,373,393). Amongst cases that had missing prehospital laboratory data of bilirubin, creatinine, and platelet, 83.7%, 85.8%, and 89.8% had at least one normal hospital laboratory result during hospital stay. GCS, Glasgow Coma Scale.

**Supplementary Table 4. The age-sex structure of the reference Hong Kong’s year 2008 adult population when calculating standardized incidence and mortality of sepsis**

| **Sex** | **Age Group**  **(years)** | **Reference:**  **2008 Year-end Hong Kong Population ('000)** |
| --- | --- | --- |
| Male | 18 - 29 | 543.3 |
| Male | 30 - 39 | 467.5 |
| Male | 40 - 49 | 586.5 |
| Male | 50 - 59 | 530.0 |
| Male | 60 - 69 | 280.3 |
| Male | 70 - 79 | 210.7 |
| Male | ≥ 80 | 86.6 |
| Female | 18 - 29 | 624.1 |
| Female | 30 - 39 | 640.7 |
| Female | 40 - 49 | 689.1 |
| Female | 50 - 59 | 538.3 |
| Female | 60 - 69 | 262.5 |
| Female | 70 - 79 | 223.6 |
| Female | ≥ 80 | 149.3 |

**Supplementary Table 5. Trends in incidence and mortality of sepsis, all infection and all hospital episodes between 2009–2018**

|  |  | **2009**  **(per 100,000)** | **2018**  **(per 100,000)** | **Relative change from 2009–2018** | **Relative annual change**  **[95% CI]** | **Adjusted R^2^** | ***p*** |
| --- | --- | --- | --- | --- | --- | --- | --- |
| **Crude Incidence** | **Sepsis** | 636 | 942 | +48.0% | +5.0% [4.1, 6.0%] | 0.94 | < 0.001 |
|  | **All Infection** | 3,198 | 4,453 | +39.2% | +4.2% [3.6, 4.7%] | 0.97 | < 0.001 |
|  | **All Hospital Episodes** | 19,188 | 24,167 | +25.9% | +2.6% [2.3, 2.9%] | 0.97 | < 0.001 |
| **Standardized Incidence** | **Sepsis** | 623 | 756 | +21.5% | +2.8% [2.0, 3.7%] | 0.87 | < 0.001 |
|  | **All Infection** | 3,136 | 3,710 | +18.3% | +2.4% [1.8, 2.9%] | 0.92 | < 0.001 |
|  | **All Hospital Episodes** | 18,931 | 21,256 | +12.3% | +1.4% [1.1, 1.6%] | 0.95 | < 0.001 |
| **Crude Mortality** | **Sepsis** | 146 | 203 | +39.0% | +4.5% [3.5, 5.4%] | 0.93 | < 0.001 |
|  | **All Infection** | 308 | 393 | +27.9% | +3.4% [2.6, 4.1%] | 0.92 | < 0.001 |
|  | **All Hospital Episodes** | 573 | 574 | +0.2% | +0.4% [-0.1, 0.9%] | 0.16 | 0.13 |
| **Standardized Mortality** | **Sepsis** | 142 | 156 | +9.6% | +1.9% [0.9, 2.8%] | 0.69 | 0.002 |
|  | **All Infection** | 300 | 300 | +0.2% | +0.7% [-0.1, 1.6%] | 0.26 | 0.08 |
|  | **All Hospital Episodes** | 558 | 440 | -21.2% | -2.1% [-2.7, -1.6%] | 0.90 | < 0.001 |

Sepsis estimates based on the primary EHR sepsis surveillance definition. Crude incidence and mortality estimates for sepsis, all infection, and all hospital episodes were calculated by dividing the total number of cases or deaths by the total Hong Kong year-end resident population for each calendar year. Exponential regression was used to model relative annual change in incidence and mortality of sepsis, all infection and all hospital episodes. CI, confidence interval.

**Supplementary Table 6. Trends in incidence of sepsis and all infection amongst all hospital episodes between 2009–2018**

|  | **2009, %** | **2018, %** | **Relative change from 2009–2018** | **Relative annual change [95% CI]** | **Adjusted R^2^** | ***p*** |
| --- | --- | --- | --- | --- | --- | --- |
| **Sepsis** | 3.3 | 3.9 | +17.5% | +2.4% [1.4, 3.3%] | 0.78 | < 0.001 |
| **All Infection** | 16.7 | 18.4 | +10.6% | +1.5% [0.9, 2.1%] | 0.80 | < 0.001 |

Exponential regression was used to model relative annual change in the proportion of sepsis and all infection amongst all hospital episodes. CI, confidence interval.

**Supplementary Table 7. Sepsis trends between 2009–2018 using different surveillance methods**

|  | **Methods** | **2009**  **(per 100,000)** | **2018**  **(per 100,000)** | **Relative change from 2009–2018** | **Relative annual change [95% CI]** | **Adjusted R^2^** | ***p*** |
| --- | --- | --- | --- | --- | --- | --- | --- |
| **Standardized Incidence** | **Primary EHR** | 623 | 756 | +21.5% | +2.8% [2.0, 3.7%] | 0.87 | < 0.001 |
|  | **Implicit** | 151 | 115 | -23.8% | -2.9% [-4.2, -1.6%] | 0.74 | < 0.001 |
|  | **Explicit** | 44 | 31 | -30.1% | -4.0% [-5.8, -2.2%] | 0.73 | 0.001 |
|  | **Martin** | 204 | 206 | +0.8% | +0.4% [-1.2, 2.0%] | -- | 0.60 |
|  | **Local sepsis codes** | 235 | 208 | -11.2% | -1.2% [-2.6, 0.1%] | 0.27 | 0.07 |
|  | **No prehospital SOFA** | 1,320 | 1,560 | +18.2% | +2.3% [1.7, 2.9%] | 0.91 | < 0.001 |
|  | **Only objective data** | 504 | 798 | +58.3% | +6.4% [5.1, 7.8%] | 0.93 | < 0.001 |
| **Standardized Mortality** | **Primary EHR** | 142 | 156 | +9.6% | +1.9% [0.9, 2.8%] | 0.69 | 0.002 |
|  | **Implicit** | 62 | 45 | -28.0% | -2.8% [-4.1, -1.4%] | 0.71 | 0.001 |
|  | **Explicit** | 21 | 13 | -38.1% | -4.8% [-6.9, -2.7%] | 0.74 | < 0.001 |
|  | **Martin** | 38 | 35 | -7.6% | +0.2% [-1.5, 1.9%] | -- | 0.79 |
|  | **Local sepsis codes** | 54 | 42 | -22.5% | -2.0% [-3.6, -0.5%] | 0.48 | 0.02 |
|  | **No prehospital SOFA** | 212 | 224 | +6.0% | +1.4% [0.5, 2.2%] | 0.60 | 0.005 |
|  | **Only objective data** | 104 | 151 | +44.8% | +5.6% [4.2, 7.1%] | 0.90 | < 0.001 |

Exponential regression was used to model relative annual change in standardized population sepsis incidence and mortality using different sepsis surveillance methods. Both exponential regression and linear regression for “Martin” method resulted in poor fit and negative R^2^. CI, confidence interval.

**Supplementary Table 8.** **Burden of sepsis: case fatality risks and proportions of sepsis-related deaths amongst total deaths**

|  |  | **2009, %** | **2018, %** | **Relative change from 2009–2018** | **Relative annual change [95% CI]** | **Adjusted R^2^** | ***p*** |
| --- | --- | --- | --- | --- | --- | --- | --- |
| **Case fatality risk** | **Sepsis** | 23.0 | 21.6 | -6.1% | -0.5% [-1.0, -0.1%] | 0.40 | 0.03 |
|  | **All Infection** | 9.6 | 8.8 | -8.2% | -0.8% [-1.3, -0.2%] | 0.47 | 0.02 |
|  | **All Hospital Episodes** | 3.0 | 2.4 | -20.4% | -2.2% [-2.7, -1.6%] | 0.89 | < 0.001 |
| **Proportion of sepsis-related deaths** | **All Infection** | 47.5 | 51.6 | +8.7% | +1.1% [0.6, 1.5%] | 0.76 | < 0.001 |
|  | **All Hospital Episodes** | 25.5 | 35.4 | +38.7% | +4.1% [3.5, 4.7%] | 0.96 | < 0.001 |
|  | **Hong Kong** | 20.9 | 27.8 | +32.8% | +3.9% [2.9, 4.8%] | 0.91 | < 0.001 |

Exponential regression was used to model relative annual change in case fatality risk of sepsis, all infection and all hospital episodes. It was also used to model relative annual change in proportion of sepsis-related deaths amongst Hong Kong deaths, all hospital episodes deaths and all infection deaths. CI, confidence interval.

**Supplementary Table 9. Reasons for misclassification by our primary EHR sepsis surveillance definition in the validation cohort**

|  | **Missing, %**  **n = 22** |
| --- | --- |
| **O_2_ therapy not captured** | 11 (50) |
| **Non-invasive ventilation not captured** | 5 (23) |
| **Use of vasopressor not recorded** | 1 (5) |
| **Platelet count not accurate due to transfusion** | 1 (5) |
| **Glasgow Coma Scale low due to Psychiatric illness** | 1 (5) |
| **Glasgow Coma Scale low due to drug overdose** | 1 (5) |
| **Long Term Home Oxygen not captured** | 1 (5) |
| **Not infection** | 1 (5) |

There were 22 hospital episodes which were misclassified when compared to physician consensus on presence of sepsis as gold standard in the validation cohort (n = 500). Each case was reviewed and the reason for misclassification was classified.

**Supplementary Figure 1. Study flow chart**

**
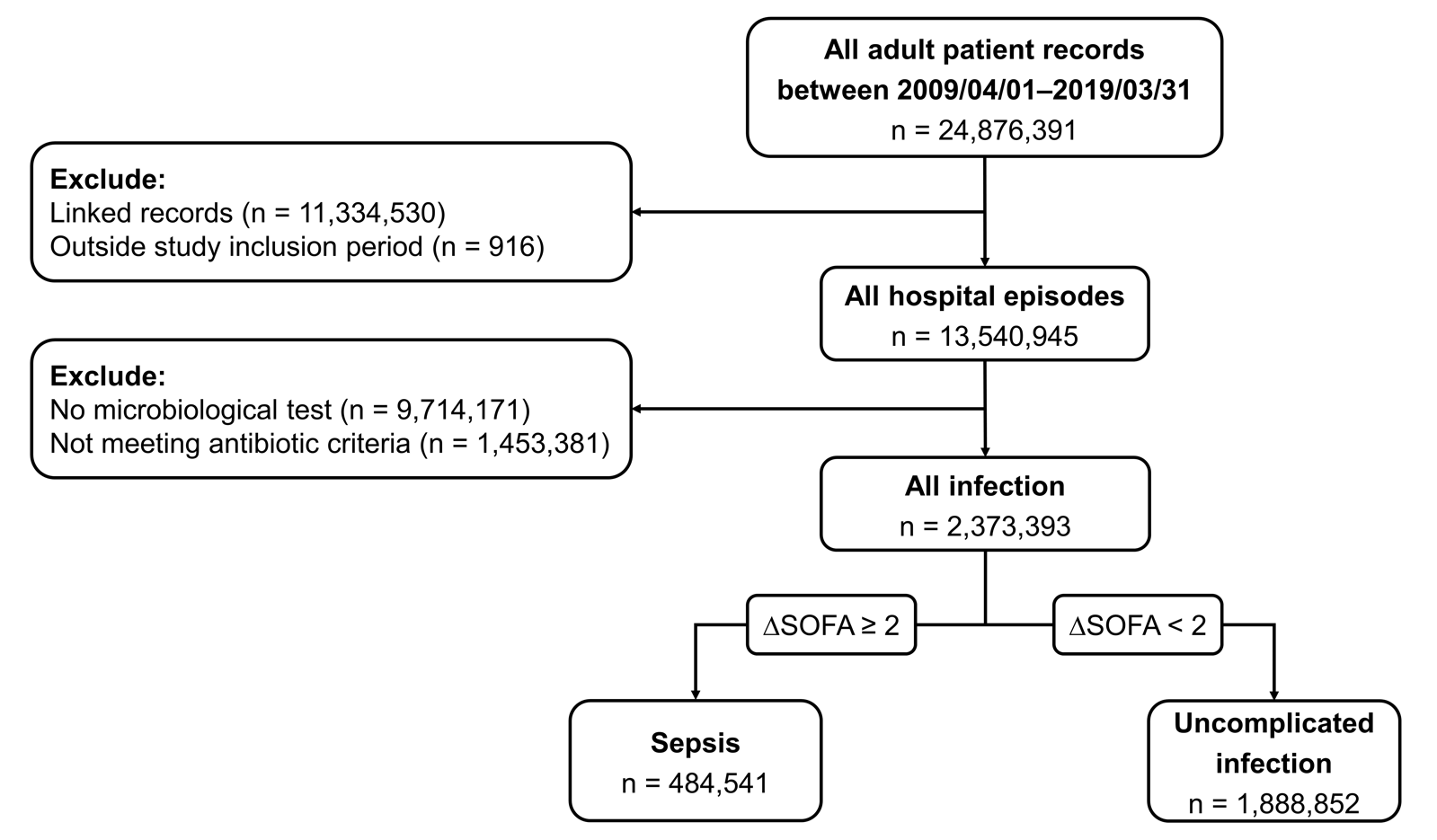
**

Study flow chart of inclusion and exclusion of patients identified from the Clinical Data Analysis and Reporting System, a population-based EHR database in Hong Kong.

**Supplementary** **Figure 2. Number of organ dysfunction and relative proportion & case fatality risk among sepsis cases**

**
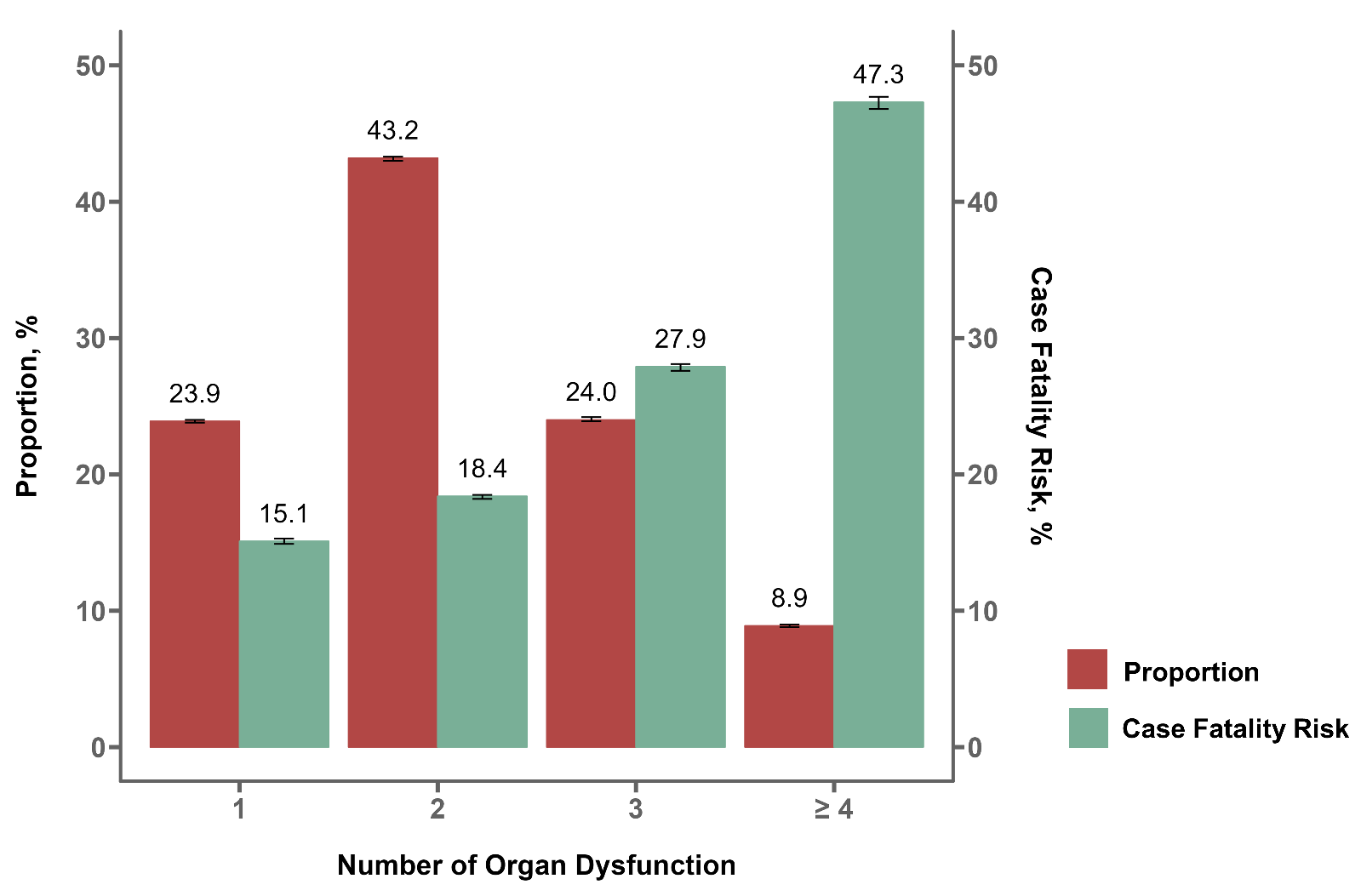
**

Sepsis cases were grouped according to the number of organ dysfunction (each estimated SOFA component ≥ 1). The proportion (red) of each group with different numbers of organ dysfunctions amongst all sepsis cases (n = 484,541) was calculated. The case fatality risk (green) of each group was calculated by dividing the number of all-cause deaths at hospital episode discharge by the total number of sepsis cases in that group. Error bars are shown as 95% confidence interval of the point estimate.

**Supplementary** **Figure 3. Trends in incidence of sepsis and all infection among all hospital episodes between 2009–2018 in Hong Kong**

**
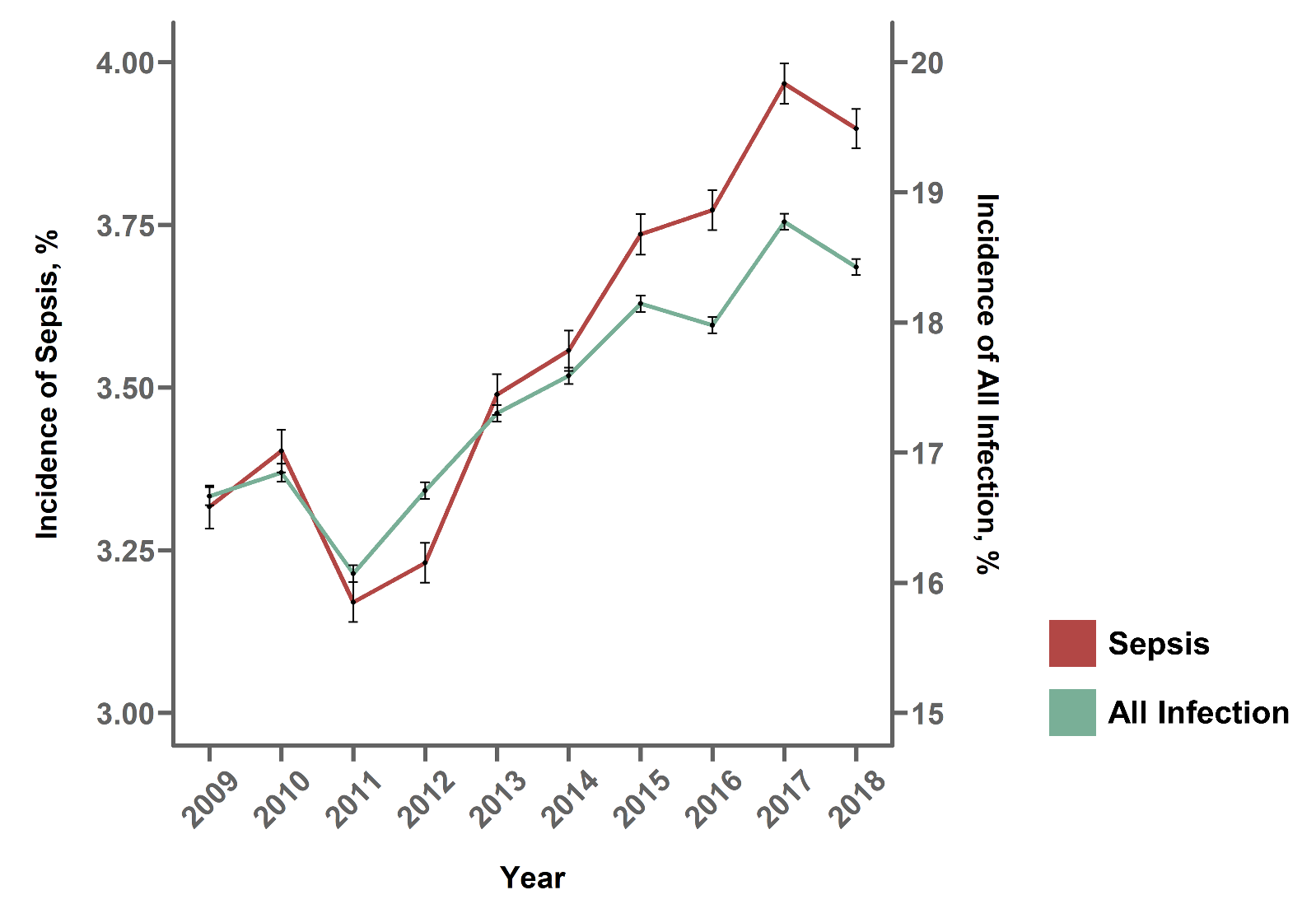
**

The incidence as calculated as the percentage of sepsis and all infection among all hospital episodes in each year. Error bars are shown as 95% confidence interval of the point estimate.

**Supplementary** **Figure 4. Comparison of case fatality risks of sepsis by age groups between 2009–2018 in Hong Kong**

**
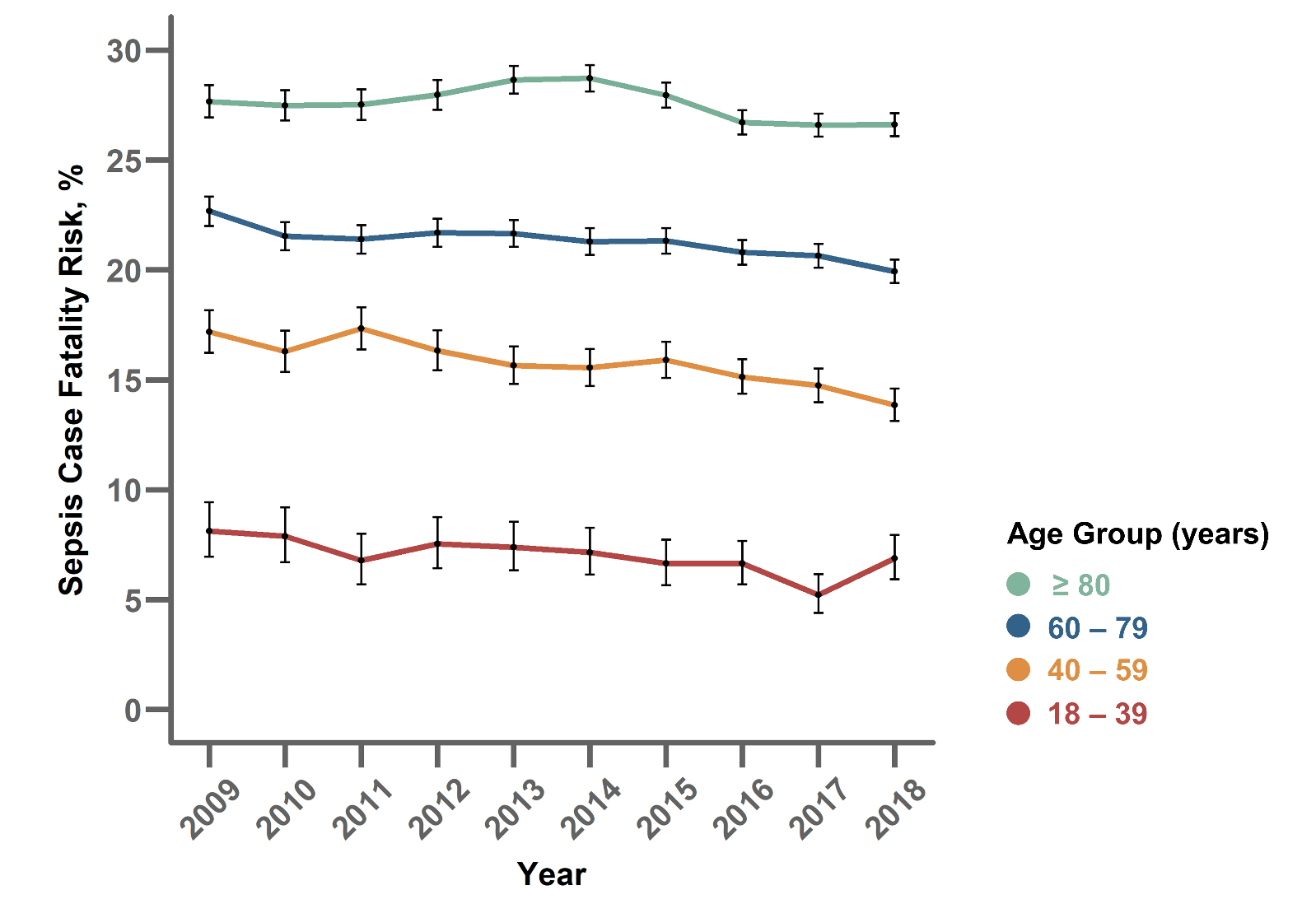
**

Trend in case fatality risk of sepsis cases stratified by age groups. Error bars are shown as 95% confidence interval of the point estimate.

**Appendix 1: Excluded microbiological tests culture type in Hong Kong population-based EHR**

| **Excluded microbiological tests culture type** |
| --- |
| Not performed |
| Culture, MRSA Screen |
| Culture, MDRA |
| Culture, VRE |
| Culture, CPE |
| Culture, MRPA |
| CRE Targeted Screening on Admission |
| VRE High Risk Screening on Haemodialysis |
| Culture, VISA |
| Culture, ESBL Screening |
| CRAB Screening |
| Culture, MSSA Screen |
| Culture, Environmental |
| Culture, Candida auris |
| Culture, Water |
| Culture, Blood Product |
| Culture, VRE (QEH pan VRE screening project 2013) |

**Appendix 2: Codes used in central nervous system and renal of prehospital/hospital SOFA score calculation**

| **Score of 2 for Central Nervous System** | **Score of 4 for Renal** |
| --- | --- |
| Diagnostic code of  Dementia (294.8:1)  Vascular dementia of acute onset (290.40:8)  Vascular dementia (290.40:11)  Dementia Alzheimer late onset (290.0:3)  Alzheimer's disease (331.0:0)  Acute vascular dementia hallucinat (290.40:13)  Dementia in Alzheimer's disease (290.10:5)  Multiinfarct dementia (290.40:5)  Dementia Alzheimer's disease (290.0:2)  Senile dementia (290.0:0)  Dementia - Lewy body disease (294.1:27)  Delirium superimposed on dementia (290.11:3)  Vascu dementia w behavior disturb (290.40:1)  Aty/ mix Alzhe dementia no add sym (290.10:13)  Dementia with depressive symptom (294.8:7)  Alzheimer disease with late onset (331.0:2)  Arteriosclerotic dementia (290.40:0)  Dementia - vitamin B12 deficiency (294.1:19)  Dementia - norm pres hydrocephalus (294.1:23)  Dementia in Parkinson's disease (294.1:3)  Vascular dementia w delirium (290.41:1)  Vasc dementia+ delusion+ beha dist (290.42:1)  Alcohol-induced dementia (291.2:0)  Vascular dementia, uncomplicated (290.40:2)  Alzheimer late onset w delusion (290.20:2)  Post infarct dementia (290.40:4)  Uncomplic Alzhe late onset+ behav (290.0:1)  Senile dementia w delirium (290.3:0)  Dement Alzhei late+ delus+ beh (290.20:1)  Acute vascular dementia mix sympto (290.40:14)  Subcortical vascular dementia (290.40:9)  Vascular dementia+ mixed symptoms (290.40:7)  Dementia with mixed symptoms (294.8:8)  Late Alzheimer dementia mix symp (290.0:7)  Mental retardation (319:0)  Dementia in early Alzheimer (290.10:6)  Dementia related to syphilis (094.1:5)  Dementia related to syphilis (294.1:15)  Mild mental retardation (317:0)  Dementia (290.0:4)  Alcoholic dementia (291.2:1)  Dement Alzheimer atypi/ mix type (290.8:1)  Fronto-temporal dementia (294.1:28)  Mild MR sig impair beh req att/ tx (317:2)  Acute vascul dementia depressive (290.43:3)  Vascu dementia predom depress (290.43:2)  Dementia - nonmedic subs poisoning (294.1:33)  Senile dementia w depress feature (290.21:0)  Subcort vas dementia hallucinatory (290.40:19)  Acute vascular dementia delusional (290.42:3)  Impaired cognition (799.8:34)  Mild cognitive disorder (294.9:4)  Alzheimer disease with early onset (331.0:1)  Alzheimer dementia delusional (290.10:10)  Mix cortical subcort vas dementia (290.40:10)  Arteriosclerot dementia w delirium (290.41:0)  Dementia in neurosyphilis (294.1:12)  Dementia due to medical condition (294.1:1)  Dementia, organic brain syndrome (294.8:2)  Severe mental retardation (318.1:0)  Sever MR sig impair behav att/ tx (318.1:2)  Presenile dementia (290.10:0)  Alzheimer dementia no add symptom (290.10:11)  Dementia related to epilepsy (294.1:25)  Acute vascular dementia no add sym (290.40:12)  Presenile dementia w delirium (290.11:0)  Dementia head trauma (294.1:2)  Cognitive disorder (294.9:1)  Vascular dementia hallucination (290.40:6)  Dement Alzheim late+ delir+ beh (290.3:1)  Dementia no add symptoms (294.8:4)  Dement Alzh early depress+ beh (290.13:1)  Delir not superimpose on dementia (293.0:1)  Alzheimer dementia depressive (290.13:5)  Pick's disease (331.1:0)  Dementia in Pick's disease (290.10:4)  Dementia with delusional symptom (294.8:5)  Alzheime early onset+ delir+ beha (290.11:1)  Subcort vas dementia mixed symptom (290.40:20)  Alzheimer's early onset w delusion (290.12:999)  Dementia in Huntington's disease (294.1:4)  Dementia related to hypothyroidism (294.1:17)  Dement Alzhe early delus+ behav (290.12:998)  Profound mental retardation (318.2:0)  Dementia related to neoplasia (294.1:16)  Cort+ subcort vas dementia mix sym (290.40:24)  Vascular dementia no add sym (290.40:25)  Vascular dementia predo delusion (290.42:2)  Multi infarct dementia no add sym (290.40:15)  Early Alzheime dementia depressive (290.13:2)  Dementia in other disease (294.1:0)  Presenile dementia w delusion (290.12:0)  Cognitive function symptom/sign (780.9:4)  Multi infarct dementia depressive (290.43:4)  Vasc dementia+ depress+ behav dist (290.43:1)  Post traumatic dementia (294.1:7)  Dement Alzheimer late+ depress (290.21:2)  Uremic dementia (294.1:30)  Senile dementia w delusion feature (290.20:0)  Dementia in spec dis mixed symptom (294.1:45)  Dementia Alzheimer early w delir (290.11:2)  Uncomp presenile dementia, beh dis (290.10:3)  Multi infarct dementia mix symps (290.40:17)  Multi infarct dementia hallucinat (290.40:16)  Alzheimer late onset w delirium (290.3:2)  Dement Alzhe late depress+ beh (290.21:1)  Dementia in Parkinson no add sym (294.1:37)  Dementia w hallucinatory symptom (294.8:6)  Dementia in Parkinson mixed sym (294.1:41)  Dementia relate to cerebral anoxia (294.1:26)  Dementia due to HIV disease (294.1:6)  Alzheimer dementia mix symptoms (290.10:16)  Late Alzheime dementia no add sym (290.0:5)  Subcort vas dementia no add sym (290.40:18)  Multi infarct dementia delusional (290.42:4)  Presenile dementia w depress featu (290.13:0)  Late Alzheimer dementia hallucinat (290.0:6)  Dementia related to hypercalcemia (294.1:21)  Presen dement Alz early+ deli+ beh (290.12:1)  Early Alzheime dementia depressive (290.13:6)  Early Alzheimer dementia mix symp (290.10:9)  Subcort vascu dementia depressive (290.43:5)  Dementia due to multiple causes (294.1:5)  Dementia in Parkinson delusional (294.1:38)  Dementia due to CJD (290.10:1)  Aty/ mix Alzhe dementia depressive (290.13:4)  Dementia - vitamin B12 deficiency (266.2:5)  Dementia in neurosyphilis (094.1:2)  Aty/ mix Alzhe dementia mix sympto (290.10:12)  Alzheimer dementia hallucinatory (290.10:15)  Dementia in Parkinson hallu sym (294.1:39)  Dementia in specifi dis delusional (294.1:43)  Dementia paralytica juvenilis (294.1:11)  Dementia - multiple sclerosis (294.1:24)  Early Alzheime dementia no add sym (290.10:20)  Aty/ mix Alzheim dementia delusion (290.12:4)  Alzheimer's dis w dementia (290.9:1)  Dementia- niacin deficien pellagra (294.1:18)  Dementia in specifi dis depressive (294.1:46)  Dementia related to drug poisoning (294.1:32) | Procedure code of  Hemodialysis for chronic care (39.95:8)  Intermittent peritoneal dialysis (54.98:2)  Create cutaneoperitoneal fistula (54.93:0)  Temp vasc access creation (38.95:1)  Continuous VV hemofiltration (39.95:6)  Hemofiltration/ hemodiafiltration (39.95:1)  Dialysis encounter (V56.8:0)  Renal dialysis encounter (V56.0:0)  Acute haemodialysis (39.95:9)  Hemodialysis temporary (39.95:2)  CAPD peritonitis (996.68:1)  Hemodialysis (39.95:0)  Venous cath for renal dialysis (38.95:0)  Peritoneal dialysis (54.98:0)  HD catheter insertion (38.95:2)  Acute haemodiafiltration (39.95:10)  Insert Tenckhoff catheter (54.93:1)  Abnormal reaction-kidney dialysis (E879.1:0)  Tenckhoff cath insert, laparoscopy (54.93:4)  Hemodialysis for acute care (39.95:15)  Continuous ambulatory PD (54.98:3)  CAPD training (54.98:1)  CVVHD (39.95:5)  PD transfer set change (97.82:4)  Creation of arteriovenous fistula (39.27:2)  Change CAPD transfer set (97.82:3)  Dialysis exit site infection (998.59:12)  Tenckhoff catheter cuff shaving (54.99:5)  Automated peritoneal dialysis (54.98:4)  Blocked Tenckhoff catheter (996.59:3)  Admitted for HD (V56.0:1)  Admitted for CAPD training (V65.49:1)  AVG creation (39.27:3)  Leaking Tenckhoff catheter (996.59:4)  Admitted for CAPD (V56.8:2)  Acute hemofiltration (39.95:7)  Displaced Tenckhoff catheter (996.59:5)  Tenckhoff catheter removal (54.90:1)  Flushing of Tenckhoff catheter (96.58:1)  Reinsertion of Tenckhoff catheter (54.93:2)  AVG, declotting (39.44:1)  Revis AV shunt for renal dialysis (39.42:0)  Cx of renal dialysis (E879.1:1)  Hemodiafiltration for chronic care (39.95:12)  Vasc access complica- d/t dialysis (996.1:5)  Infected Tenckhoff catheter (996.68:2)  Hemodiafiltration for acute care (39.95:17)  Subcutaneous leak dialysis relate (996.59:9)  Tenckhoff cath reposit-laparoscopy (54.90:3)  Haemofiltration for acute care (39.95:16)  Replace Tenckhoff cath (54.90:2)  Hemofiltration for chronic care (39.95:11)  Blocked arteriovenous fistula (996.1:1)  Infection PD catheter (996.68:0)  Tenckhoff catheter,repositioning (54.90:4)  Hemodiafiltration (39.95:14)  PD catheter malfunction (996.59:8)  AVF, declotting (39.44:2)  Dialysis disequilibrium syndrome (276.9:2)  Hemoperitoneum related to dialysis (568.81:4) |

**Appendix 3: “Implicit” sepsis surveillance definition (defined as: ≥ 1 infection code and 1 organ dysfunction code or explicit codes for severe sepsis or septic shock)**

| **ICD-9-CM Codes^*^** **for severe sepsis/SIRS, infect with organ failure, and septic shock** | **Description** |
| --- | --- |
| 780.9:29^1^ | SIRS, infect with organ failure |
| 785.59:2^2^ | Septic shock |
|  | |
| **ICD-9-CM Codes^*^** **used to identify bacterial or fungal infection** | **Description** |
| 001 | Cholera |
| 002 | Typhoid/paratyphoid fever |
| 003 | Other salmonella infection |
| 004 | Shigellosis |
| 005 | Other food poisoning |
| 008 | Intestinal infection not otherwise classified |
| 009 | Ill-defined intestinal infection |
| 010 | Primary tuberculosis infection |
| 011 | Pulmonary tuberculosis |
| 012 | Other respiratory tuberculosis |
| 013 | Central nervous system tuberculosis |
| 014 | Intestinal tuberculosis |
| 015 | Tuberculosis of bone and joint |
| 016 | Genitourinary tuberculosis |
| 017 | Tuberculosis not otherwise classified |
| 018 | Miliary tuberculosis |
| 020 | Plague |
| 021 | Tularemia |
| 022 | Anthrax |
| 023 | Brucellosis |
| 024 | Glanders |
| 025 | Melioidosis |
| 026 | Rat-bite fever |
| 027 | Other bacterial zoonoses |
| 030 | Leprosy |
| 031 | Other mycobacterial disease |
| 032 | Diphtheria |
| 033 | Whooping cough |
| 034 | Streptococcal throat/scarlet fever |
| 035 | Erysipelas |
| 036 | Meningococcal infection |
| 037 | Tetanus |
| 038 | Septicemia |
| 039 | Actinomycotic infections |
| 040 | Other bacterial diseases |
| 041 | Bacterial infection in other diseases not otherwise specified |
| 090 | Congenital syphilis |
| 091 | Early symptomatic syphilis |
| 092 | Early syphilis latent |
| 093 | Cardiovascular syphilis |
| 094 | neurosyphilis |
| 095 | Other late symptomatic syphilis |
| 096 | Late syphilis latent |
| 097 | Other and unspecified syphilis |
| 098 | Gonococcal infections |
| 100 | Leptosprosis |
| 101 | Vincent’s angina |
| 102 | Yaws |
| 103 | Pinta |
| 104 | Other spirochetal infection |
| 110 | Dermatophytosis |
| 111 | Dermatomycosis not otherwise classified or specified |
| 112 | Candidiasis |
| 114 | Coccidioidomycosis |
| 115 | Histoplasmosis |
| 116 | Blastomycotic infection |
| 117 | Other mycoses |
| 118 | Opportunistic mycoses |
| 320 | Bacterial meningitis |
| 322 | Meningitis, unspecified |
| 324 | Central nervous system abscess |
| 325 | Phlebitis of intracranial sinus |
| 420 | Acute pericarditis |
| 421 | Acute or subacute endocarditis |
| 451 | Thrombophlebitis |
| 461 | Acute sinusitis |
| 462 | Acute pharyngitis |
| 463 | Acute tonsillitis |
| 464 | Acute laryngitis/tracheitis |
| 465 | Acute upper respiratory infection of multiple sites/not otherwise specified |
| 481 | Pneumococcal pneumonia |
| 482 | Other bacterial pneumonia |
| 485 | Bronchopneumonia with organism not otherwise specified |
| 486 | Pneumonia, organism not otherwise specified |
| 491.21 | Acute exacerbation of obstructive chronic bronchitis |
| 494 | Bronchiectasis |
| 510 | Empyema |
| 513 | Lung/mediastinum abscess |
| 540 | Acute appendicitis |
| 541 | Appendicitis not otherwise specified |
| 542 | Other appendicitis |
| 562.01 | Diverticulitis of small intestine without hemorrhage |
| 562.03 | Diverticulitis of small intestine with hemorrhage |
| 562.11 | Diverticulitis of colon without hemorrhage |
| 562.13 | Diverticulitis of colon with hemorrhage |
| 556 | Anal and rectal abscess |
| 567 | Peritonitis |
| 569.5 | Intestinal abscess |
| 569.83 | Perforation of intestine |
| 572 | Abscess of liver |
| 572.1 | Portal pyemia |
| 575.0 | Acute cholecystitis |
| 590 | Kidney infection |
| 597 | Urethritis/urethral syndrome |
| 599.0 | Urinary tract infection not otherwise specified |
| 601 | Prostatic inflammation |
| 614 | Female pelvic inflammation disease |
| 615 | Uterine inflammatory disease |
| 616 | Other female genital inflammation |
| 681 | Cellulitis, finger/toe |
| 682 | Other cellulitis or abscess |
| 683 | Acute lymphadenitis |
| 686 | Other local skin infection |
| 711.0 | Pyogenic arthritis |
| 730 | Osteomyelitis |
| 790.7 | Bacteremia |
| 996.6 | Infection or inflammation of device/graft |
| 998.5 | Postoperative infection |
| 999.3 | Infectious complication of medical care not otherwise classified |
|  | |
| **ICD-9-CM Codes^*^** **used to identify organ dysfunction** | **Description** |
| 785.5 | Shock without trauma |
| 458 | Hypotension |
| 96.7 | Mechanical ventilation |
| 348.3 | Encephalopathy |
| 293 | Transient organic psychosis |
| 348.1 | Anoxic brain damage |
| 287.4 | Secondary thrombocytopenia |
| 287.5 | Thrombocytopenia, unspecified |
| 286.9 | Other/unspecified coagulation defect |
| 286.6 | Defibrination syndrome |
| 570 | Acute and subacute necrosis of liver |
| 573.4 | Hepatic infarction |
| 584 | Acute renal failure |

Codes were modified ICD-9-CM codes in Clinical Data Analysis and Reporting System (population public electronic health record database in Hong Kong).

^1^Corresponding to 995.92 Severe Sepsis (ICD-9-CM)

^2^Corresponding to 785.52 Septic shock (ICD-9-CM)

**Appendix 4: “Explicit” sepsis surveillance definition (defined as: explicit codes for severe sepsis or septic shock)**

| **ICD-9-CM^*^ Codes** **for severe sepsis, and septic shock** | **Description** |
| --- | --- |
| 780.9:29^1^ | SIRS, infect with organ failure |
| 785.59:2^2^ | Septic shock |

Codes were modified ICD-9-CM codes in Clinical Data Analysis and Reporting System (population public electronic health record database in Hong Kong).

^1^Corresponding to 995.92 Severe Sepsis (ICD-9-CM)

^2^Corresponding to 785.52 Septic shock (ICD-9-CM)

**Appendix 5: “Martin” sepsis surveillance definition (defined as: codes for septicemia or disseminated infection)**

| **ICD-9-CM Codes** **for sepsis** | **Description** |
| --- | --- |
| 038 | Septicemia |
| 020.2^1^ | Septicaemic plague |
| 790.7 | Bacteremia |
| 117.9 | Disseminated fungal infection |
| 112.5 | Disseminated candida infection |
| 112.81 | Disseminated fungal endocarditis |

Codes were modified ICD-9-CM codes in Clinical Data Analysis and Reporting System (population public electronic health record database in Hong Kong).

^1^Corresponding to 020.0 Septicemic (ICD-9-CM)

**Appendix 6: “Local sepsis codes” sepsis surveillance definition (defined as: any sepsis-related code in CDARS)**

| **Local ICD-9-CM Codes** **for sepsis in Hong Kong centralized EHR** | **Description** |
| --- | --- |
| 038.49:2 | Acinetobacter septicemia |
| 039.9:4 | Actinomycotic sepsis |
| 039.9:5 | Actinomycotic septicemia |
| 038.3:1 | Anaerobic sepsis |
| 038.3:0 | Anaerobic septicemia |
| 112.5:1 | Candidal sepsis |
| 038.9:1 | Clinical sepsis |
| 038.1:3 | Coagulase -ve staphy septicemia |
| 038.42:0 | E. coli septicemia |
| 038.49:1 | Enterobacter septicemia |
| 027.1:5 | Erysipelothrix septicemia |
| 117.9:5 | Fungal septicemia |
| 038.40:1 | Gram negative sepsis |
| 038.40:0 | Gram negative septicemia |
| 038.0:7 | Group A streptococcal septicemia |
| 038.0:9 | Group B streptococcal septicemia |
| 038.0:8 | Group D streptococcal septicemia |
| 038.41:2 | Hemophilus flu sepsis |
| 038.41:0 | Hemophilus flu septicemia |
| 038.41:1 | HIB septicemia |
| 038.9:2 | Intra-abdominal sepsis |
| 038.49:4 | Klebsiella pneumoniae septicemia |
| 038.49:3 | Klebsiella septicemia |
| 027.0:4 | Listerial sepsis |
| 027.0:5 | Listerial septicemia |
| 038.1:4 | Methicillin resist SA septicemia |
| 038.1:1 | Multi resist staphylo septicemia |
| 038.2:1 | Pneumococcal sepsis |
| 038.43:0 | Pseudomonas septicemia |
| 670.04:2 | Puerperal sepsis |
| 003.1:2 | Recurrent salmonella septicemia |
| 003.1:1 | Salmonella sepsis |
| 003.1:0 | Salmonella septicemia |
| 038.9:5 | Sepsis |
| 038.49:5 | Sepsis due to Moraxella infection |
| 998.59:11 | Sepsis, post-op complication |
| 785.59:2 | Septic shock |
| 648.93:6 | Septic shock for antepartum care |
| 648.91:1 | Septic shock, delivery episode |
| 038.0:2 | Septicaemia due to VRE |
| 998.59:10 | Septicaemia, post-op |
| 038.9:0 | Septicemia |
| 038.44:0 | Serratia septicemia |
| 038.1:5 | Staph aureus sepsis |
| 038.1:2 | Staph aureus septicemia |
| 038.1:6 | Staphylococcal sepsis |
| 038.1:0 | Staphylococcal septicemia |
| 038.2:0 | Strepto pneumococcal septicemia |
| 038.0:3 | Streptococcal sepsis |
| 038.0:4 | Streptococcal sepsis, group A |
| 038.0:5 | Streptococcal sepsis, group B |
| 038.0:6 | Streptococcal sepsis, group D |
| 038.0:0 | Streptococcal septicemia |
| 038.0:1 | Streptococcus suis septicemia |

**Appendix 7: Physician review of validation cohort**

Two physicians blinded to the primary EHR method independently reviewed the medical records to determine presence of sepsis (Sepsis-3 criteria) in each of the 500 selected hospital episodes. They reviewed clinical notes that contained inpatient and outpatient records on history, examination, working diagnoses, discharge summaries and outcomes. Hospital admission vitals including blood pressure, heart rate, SpO2 were sometimes documented in examination findings but daily routine vital observations were not available for review. All laboratory, microbiology imaging results were reviewed. Antimicrobial prescription and other medication orders were also reviewed. Physicians were given instructions to determine the presence of sepsis at time of index culture date. Disagreements were resolved by discussion and arbitrated by a third clinician if agreement could not be reached.

**Appendix 8: Site of infection**

| **Site of infection** | **Culture Specimen Type** | **Infection diagnosis code in Hong Kong centralized EHR** |
| --- | --- | --- |
| **Respiratory** | Sputum | Abscess of lung (513.0:0) |
|  | Pleural fluid | Abscess of lung with pneumonia (513.0:2) |
|  | Bronchoalveolar lavage | Abscess of mediastinum (513.1:0) |
|  | Endotracheal aspirate | Ac bronchiol d/t infect org (466.19:0) |
|  | Tracheal aspirate | Acute bronchiolitis by RSV (466.11:0) |
|  | Bronchial aspirate | Acute bronchiolitis due to hMPV (466.19:1) |
|  | Pleural fluid, Right | Acute bronchitis (466.0:0) |
|  | Nasopharyngeal aspirate | Acute bronchitis by coxsackievirus (466.0:7) |
|  | Bronchial brushing | Acute bronchitis by hemophilus (466.0:5) |
|  | Pleural fluid, Left | Acute bronchitis by parainfluenza (466.0:8) |
|  | Chest drain fluid | Acute bronchitis by rhinovirus (466.0:10) |
|  | Endotracheal tube | Acute bronchitis by RSV (466.0:9) |
|  | Pleural biopsy | Acute bronchitis by streptococcus (466.0:6) |
|  | Bronchial trap | Acute mycoplasmal bronchitis (466.0:4) |
|  | Tracheostomy aspirate | Acute URTI (465.9:0) |
|  | Bronchial washing | Adenoviral pneumonia (480.0:0) |
|  | Tracheostomy tube | Amebic lung abscess (006.4:0) |
|  | Lung biopsy | Aspergillosis (117.3:0) |
|  | Transtracheal aspirate | Aspiration pneumonia (507.0:1) |
|  | Transbronchial aspirate | Aspiration pneumonia, post-op (507.0:3) |
|  | Bronchial biopsy | Aspiration pneumonitis (507.0:4) |
|  | Tracheostomy wound swab | Bronchio obliter organis pneumonia (516.8:1) |
|  |  | Bronchitis (490:0) |
|  |  | Candidal pneumonia (112.4:1) |
|  |  | Candidiasis of lung (112.4:0) |
|  |  | Chest infection (519.8:1) |
|  |  | Chlamydia psittaci infection (073.9:1) |
|  |  | CMV pneumonia (078.5:1) |
|  |  | Congenital pneumonia (770.0:0) |
|  |  | COPD w acute lower resp infection (496:3) |
|  |  | Delivered, PP pulmonary infection (648.92:803) |
|  |  | Empyema (510.9:0) |
|  |  | Empyema thoracis (510.9:1) |
|  |  | Empyema with fistula (510.0:0) |
|  |  | Foreign body pneumonia (507.8:1) |
|  |  | Fungal pneumonia (117.9:2) |
|  |  | Gangrenous pneumonia (513.0:3) |
|  |  | H1N1 - human swine flu (487.1:11) |
|  |  | Haem influenzae type B infection (041.5:2) |
|  |  | Hemophilus flu sepsis (038.41:2) |
|  |  | Hemophilus flu septicemia (038.41:0) |
|  |  | Hemophilus infection (041.85:9) |
|  |  | Hemophilus influenza infection (041.5:0) |
|  |  | Hemophilus pneumonia (482.2:0) |
|  |  | Human metapneumovirus infection (079.89:8) |
|  |  | Human metapneumovirus pneumonia (480.8:3) |
|  |  | Infection due to coronavirus (079.89:3) |
|  |  | Influenza (487.8:0) |
|  |  | Influenza A (487.1:3) |
|  |  | Influenza A H1N1 viral infection (487.1:9) |
|  |  | Influenza A H3N2 (487.1:7) |
|  |  | Influenza A H5 (487.1:1) |
|  |  | Influenza A H5N1 viral infection (487.1:2) |
|  |  | Influenza A H7 (487.1:5) |
|  |  | Influenza A H9 (487.1:6) |
|  |  | Influenza A human swine flu (487.1:10) |
|  |  | Influenza B (487.1:4) |
|  |  | Influenza by identified flu virus (487.8:3) |
|  |  | Influenza C (487.1:12) |
|  |  | Influenza like illness (079.89:11) |
|  |  | Influenza vaccination (99.52:0) |
|  |  | Influenza with GE (487.8:1) |
|  |  | Influenza with pneumonia (487.0:0) |
|  |  | Influenza, antepartum care (648.93:864) |
|  |  | Interstitial pneumonia (516.8:5) |
|  |  | Klebsiella pneumonia (482.0:0) |
|  |  | Klebsiella pneumoniae septicemia (038.49:4) |
|  |  | Late effect of respiratory TB (137.0:0) |
|  |  | Late effects of tuberculosis (137.4:0) |
|  |  | Lung mycobacterium infection (031.0:0) |
|  |  | Mycoplasma infection (041.81:0) |
|  |  | Mycoplasma pneumonia (483.0:0) |
|  |  | Parainfluenza infection (079.89:4) |
|  |  | Pneumococcal pneumonia (481:0) |
|  |  | Pneumocystis carinii infection (136.3:2) |
|  |  | Pneumonia (486:0) |
|  |  | Pneumonia due to coronavirus (480.8:1) |
|  |  | Pneumonia due to VRE (482.39:3) |
|  |  | Pneumonia E coli (482.82:0) |
|  |  | Pneumonia gram -ve bacteria (482.83:0) |
|  |  | Pneumonia in aspergillosis (484.6:0) |
|  |  | Pneumonia in cytome inclusion dis (484.1:0) |
|  |  | Pneumonia in infectious disease (136.9:1) |
|  |  | Pneumonia in infectious diseases (484.8:0) |
|  |  | Pneumonia in whooping cough (484.3:0) |
|  |  | Pneumonia strep. B (482.32:0) |
|  |  | Pneumonia streptococcus A (482.31:0) |
|  |  | Pneumonia- food/ vomitus postop (507.0:2) |
|  |  | Pneumonia, anaerobes (482.81:0) |
|  |  | Pneumonia, antepartum (648.93:863) |
|  |  | Pneumonia, Hypostatic (514:1) |
|  |  | Pneumonia, Lipoid (507.1:1) |
|  |  | Pneumonia, organism (486:999) |
|  |  | Pneumonia, postpartum (648.94:863) |
|  |  | Pneumonia, staphylococcus (482.4:0) |
|  |  | Post-operative pneumonia (486:2) |
|  |  | Pseudomonas pneumonia (482.1:0) |
|  |  | Pulmonary actinomycosis (039.1:0) |
|  |  | Pulmonary coccidioidomycosis (114.5:0) |
|  |  | Pulmonary cryptococcosis (117.5:3) |
|  |  | Pulmonary TB - exam unknown (011.92:0) |
|  |  | Pulmonary TB by culture (011.94:0) |
|  |  | Pulmonary TB by histology (011.95:0) |
|  |  | Pulmonary TB by micro exam (011.93:0) |
|  |  | Pulmonary TB confirmed (011.96:0) |
|  |  | Pulmonary tuberculosis (011.90:0) |
|  |  | Resp syncytial virus infection (079.6:0) |
|  |  | Respiratory infection (519.8:5) |
|  |  | Respiratory infection, antepartum (648.93:803) |
|  |  | Respiratory TB, antepartum care (647.33:802) |
|  |  | Rhinovirus infection (079.3:0) |
|  |  | RSV pneumonia (480.1:0) |
|  |  | Salmonella pneumonia (003.22:0) |
|  |  | SARS with atypical pneumonia (480.8:2) |
|  |  | Strepto pneumoniae infection (041.06:0) |
|  |  | Streptococcal pneumonia (482.30:0) |
|  |  | Streptococcu suis bronchopneumonia (482.39:1) |
|  |  | Streptococcu suis pneumonia (482.39:2) |
|  |  | TB endobronchus (012.20:1) |
|  |  | TB lung cavitation by culture (011.24:0) |
|  |  | TB lung cavitation by histology (011.25:0) |
|  |  | TB lung cavitation by micro exam (011.23:0) |
|  |  | TB lung cavitation, confirmed (011.26:0) |
|  |  | TB lung w cavitation (011.20:0) |
|  |  | TB Pleura (012.00:0) |
|  |  | TB pleural effusion (012.00:1) |
|  |  | Viral pneumonia (480.8:0) |
|  |  | Viral pneumonia (480.9:0) |
|  | | |
| **Urinary** | Midstream urine | Ac pyelonephritis w medull necros (590.11:0) |
|  | Catheterized urine | Acu infect tubulointerstit nephrit (580.89:9) |
|  | Urine | Acute cystitis (595.0:0) |
|  | Catheterized urine, catheterized once | Acute hemorrhagic cystitis (595.0:1) |
|  | Early morning urine | Acute pyelonephritis (590.10:0) |
|  | Percutaneous nephrostomy urine, Right | Acute pyelonephritis post op (590.10:1) |
|  | Bag urine | Bacteriuria (791.9:4) |
|  | Catheterized urine, indwelling | Chronic obstructive pyelonephritis (590.00:2) |
|  | Percutaneous nephrostomy urine, Left | Chronic pyelonephritis (590.00:0) |
|  | Nephrostomy urine | Infection of kidney (590.9:0) |
|  | Midstream urine, dipslide | Leaking left nephrostomy tube (996.39:17) |
|  | Kidney urine, Right | Necrotizing glomerulonephritis (583.4:10) |
|  | Kidney urine, Left | Pyelonephrit in oth dis (590.81:0) |
|  | Suprapubic urine | Pyelonephritis (590.80:0) |
|  | Percutaneous nephrostomy urine | Pyelonephritis (590.80:3) |
|  | Ureteric urine, Left | Pyelonephritis, antepartum care (646.63:809) |
|  | Ureteric urine | Pyelonephritis, postoperative (590.80:5) |
|  | Ureteric urine, Right | Pyelonephritis, postpartum care (646.64:809) |
|  | Nephrostomy urine, Right | Renal / perirenal abscess (590.2:0) |
|  | Nephrostomy urine, Left | Renal Abscess (590.2:2) |
|  | Bladder urine | TB bladder - no exam (016.11:0) |
|  | Conduit urine | TB bladder by histology (016.15:0) |
|  | Kidney urine | TB bladder by micro exam (016.13:0) |
|  | Clean catched urine | TB bladder confirmed (016.16:0) |
|  | Urethral urine | TB kidney - exam unknown (016.02:0) |
|  | Catheterized urine, dipslide | TB kidney - no exam (016.01:0) |
|  | Urine, dipslide | TB kidney by culture (016.04:0) |
|  | Catheterized urine, indwelling, dipslide | TB kidney by histology (016.05:0) |
|  | Catheterized urine, catheterized once, dipslide | TB kidney confirmed (016.06:0) |
|  | Stamey's test, voided bladder 1 urine (VB1) | TB kidney micro exam (016.03:0) |
|  | Bag urine, dipslide | TB ureter - exam unknown (016.22:0) |
|  | Stamey's test, voided bladder 2 urine (VB2) | TB ureter (016.20:0) |
|  | Stamey's test, voided bladder 3 urine (VB3) | TB urinary organ - confirmed (016.36:0) |
|  |  | TB urinary organ (016.30:0) |
|  |  | TB urinary organ by culture (016.34:0) |
|  |  | TB urinary organ by histology (016.35:0) |
|  |  | TB urinary organ by micro eaxm (016.33:0) |
|  |  | Tuberculosis pyelonephritis (016.00:3) |
|  |  | Tuberculous kidney (016.00:0) |
|  |  | Urinary tract infection (599.0:0) |
|  | | |
| **Gastrointestinal** | Stool | Abdominopelvic Abscess (567.2:11) |
|  | Bile | Abscess of spleen (289.59:3) |
|  | Peritoneal swab | Acute amebic dysentery (006.0:0) |
|  | Peritoneal dialysis fluid | Acute and chronic colitis (558.9:29) |
|  | Ascitic fluid | Acute appendicitis (540.9:0) |
|  | Peritoneal fluid | Acute appendicitis+ gen peritoniti (540.0:0) |
|  | Gastric aspirate | Acute appendicitis+ perito abscess (540.1:0) |
|  | Dialysis fluid | Acute cholangitis (576.1:1) |
|  | Gall bladder bile | Acute cholecystitis (575.0:0) |
|  | Bile, PTBD | Acute duodenal ulcer w perforation (532.10:0) |
|  | Gastric lavage | Acute enterocolitis (558.9:9) |
|  | Umbilical swab | Acute gastric ulcer w perforation (531.10:0) |
|  | Bile, common bile duct | Acute hemorrhagic cholecystitis (575.0:3) |
|  | Peritoneal dialysis fluid, CAPD | Acute hemorrhagic enterocolitis (557.0:17) |
|  | Peritoneal dialysis fluid, post-dialysis | Acute hep B & delta (070.31:0) |
|  | Liver abscess | Acute hepatitis B with coma (070.20:0) |
|  | Abdominal swab | Acute hepatitis C infection (070.51:0) |
|  | Cholecystostomy drain fluid | Acute hepatitis C with coma (070.41:0) |
|  | Bile, t-tube | Acute peptic ulcer w perforation (533.10:0) |
|  | Appendicular swab | Acute peritonitis (567.9:1) |
|  | Dialysate fluid, pre-dialysis | Acute suppurative cholecystitis (575.0:4) |
|  | Suphrenic fluid | Acute viral hepatitis B infection (070.30:0) |
|  | Peritoneal catheter | Amebiasis (006.9:0) |
|  | Liver biopsy | Amebic colitis (006.9:1) |
|  | Colon biopsy | Amebic dysentery (006.9:2) |
|  | Dialysate fluid, post-dialysis | Amebic liver abscess (006.3:0) |
|  | Gastrostomy swab | Amebic nondysenteric colitis (006.2:0) |
|  | Gastric biopsy | Amebic skin ulceration (006.6:0) |
|  | Rectal biopsy | Ameboma of intestine (006.8:3) |
|  | Oesophageal biopsy | Anal & rectal abscess (566:0) |
|  |  | Anal & rectal polyp (569.0:0) |
|  |  | Anal abscess (566:8) |
|  |  | Bacterial intestinal infection (008.49:0) |
|  |  | Bile leakage from liver (573.8:4) |
|  |  | Bile leakage from PTBD (997.4:26) |
|  |  | Bile leakage from T-tube (997.4:3) |
|  |  | Bile peritonitis (567.8:1) |
|  |  | Bowel inf, vibrio parahemolyticus (008.46:4) |
|  |  | Bowel infect aeromonas hydrophila (008.46:1) |
|  |  | Bowel infect by campylobact jejuni (008.43:2) |
|  |  | Bowel infect by campylobacter coli (008.43:1) |
|  |  | Bowel infect by enteroinvas E coli (008.03:0) |
|  |  | Bowel infect by enteropatho E coli (008.01:0) |
|  |  | Bowel infection by salmonella A (003.0:1) |
|  |  | Campylobacter intestinal infection (008.43:0) |
|  |  | Candidiasis of intestine (112.85:0) |
|  |  | CAPD peritonitis (996.68:1) |
|  |  | Cecal diverticulum, perforation (562.10:1) |
|  |  | Cholangitis (576.1:0) |
|  |  | Cholecystitis (575.10:0) |
|  |  | Chronic Appendicitis (542:1) |
|  |  | Chronic duoden ulcer+ perforation (532.50:0) |
|  |  | Chronic hepatitis C infection (070.54:0) |
|  |  | Chronic peptic ulcer w perforation (533.50:0) |
|  |  | CMV hepatitis (078.5:3) |
|  |  | Diarrhea presumed infectious (009.3:0) |
|  |  | Drain appendiceal abscess (47.2:0) |
|  |  | Drain extraperit abscess percutane (54.0:6) |
|  |  | Drain intraperiton absce percutane (54.19:9) |
|  |  | Drain liver abscess, percutaneous (50.0:4) |
|  |  | Drain retroperit absces percutaneo (54.0:8) |
|  |  | Drain retroperitoneal abscess open (54.0:7) |
|  |  | Drainage extraperitoneal abscess (54.0:3) |
|  |  | Drainage intraperiton abscess open (54.19:8) |
|  |  | Drainage intraperitoneal abscess (54.19:7) |
|  |  | Drainage pelv abscess, laparotomy (70.12:2) |
|  |  | E. coli O157:H7 enteritis (008.04:1) |
|  |  | Empyema gallbladder (575.0:1) |
|  |  | Entamoeba histolytica Infection (006.9:4) |
|  |  | Enteritis (558.9:2) |
|  |  | Enteritis by small round viruses (008.64:0) |
|  |  | Enteritis due to adenovirus (008.62:0) |
|  |  | Enteritis due to astrovirus (008.66:0) |
|  |  | Enteritis due to calicivirus (008.65:0) |
|  |  | Enteritis due to enterovirus (008.67:0) |
|  |  | Enteritis due to norwalk virus (008.63:0) |
|  |  | Enteritis due to rotavirus (008.61:0) |
|  |  | Enteritis due to virus (008.69:0) |
|  |  | Enteritis/ GE presumed infectious (009.1:0) |
|  |  | Entero- arthropathy, ulcer colitis (556.9:1) |
|  |  | Fecal peritonitis (567.8:2) |
|  |  | Fungal enteritis (008.8:1) |
|  |  | Fungal peritonitis (117.9:4) |
|  |  | Gallstone w cholecystitis (574.10:0) |
|  |  | Gallstone w inflammation+ obstruct (574.11:0) |
|  |  | Gallstone with obstruction (574.21:0) |
|  |  | GB stone w acute cholecystitis (574.00:0) |
|  |  | GB stone+ acu cholecystitis& obstr (574.01:0) |
|  |  | GB& bile duct stone+ AC & obstruct (574.61:0) |
|  |  | GB& bili cal+ acu/ch cholecy+ obst (574.81:0) |
|  |  | GB& bili calcu+ acu/chr cholecysti (574.80:0) |
|  |  | GB+ bile duct calc+ acute cholecys (574.60:0) |
|  |  | GE presumed infectious origin (009.1:1) |
|  |  | GE, salmonella B (003.0:2) |
|  |  | GE, salmonella C (003.0:3) |
|  |  | GE, salmonella D (003.0:4) |
|  |  | GE, salmonella E (003.0:5) |
|  |  | Helicobacter pylori infection (041.86:0) |
|  |  | Hepatitis antigen +ve, pregnant (V02.61:2) |
|  |  | Hepatitis antigen positive (V02.61:3) |
|  |  | Hepatitis B reactivation (070.30:2) |
|  |  | Hepatitis B, antepartum care (647.63:806) |
|  |  | Hepatitis B, postpartum care (647.64:806) |
|  |  | Hepatitis C, antepartum care (647.63:807) |
|  |  | Hepatitis D infection (070.52:0) |
|  |  | Hepatitis E infection (070.53:0) |
|  |  | Hepatitis E virus infection (070.53:1) |
|  |  | Hepatitis E with coma (070.43:0) |
|  |  | Hepatitis in infectious disease (573.2:0) |
|  |  | Hepatitis in viral disease (573.1:0) |
|  |  | I&D extraperitoneal absces (54.0:5) |
|  |  | I&D of perianal abscess (49.01:1) |
|  |  | Incise perianal abscess (49.01:0) |
|  |  | Incision of perianal tissue (49.02:0) |
|  |  | Infected ascites (789.5:3) |
|  |  | Infected Tenckhoff catheter (996.68:2) |
|  |  | Infection of retroperitoneal space (567.9:4) |
|  |  | Infection PD catheter (996.68:0) |
|  |  | Infectious colitis, enteritis & GE (009.0:0) |
|  |  | Infectious diarrhea (009.2:0) |
|  |  | Infectious gastroenteritis (009.0:4) |
|  |  | Infectious peritonitis (567.0:0) |
|  |  | Infective colitis (009.0:2) |
|  |  | Infective enteritis (009.0:3) |
|  |  | Infective peritonitis (567.2:13) |
|  |  | Infective proctitis with bleeding (569.49:7) |
|  |  | Intes infec clostridium botulinum (005.1:1) |
|  |  | Intest infect, clostrid difficile (008.45:1) |
|  |  | Intest infect, enteroadhere E Coli (008.09:1) |
|  |  | Intest infection, cholera non-O139 (001.9:4) |
|  |  | Intestinal abscess (569.5:0) |
|  |  | Intestinal infect, cholera non-O1 (001.9:3) |
|  |  | Intestinal infection by anaerobes (008.46:0) |
|  |  | Intestinal infection due to E coli (008.00:0) |
|  |  | Intestinal infection, anaerobes (008.46:2) |
|  |  | Intestinal infection, cholera O1 (001.9:1) |
|  |  | Intestinal infection, clostridium (008.46:3) |
|  |  | Intestinal infection, proteus (008.3:1) |
|  |  | Intestinal malabsorption (579.9:0) |
|  |  | Intestinal obstruction (560.9:0) |
|  |  | Intestinal obstruction, postop (560.9:2) |
|  |  | Intestinal parasitism (129:0) |
|  |  | Intestinal stricture (560.9:13) |
|  |  | Intestinal strongyloidiasis (127.2:2) |
|  |  | Intestine infect gram neg bacteria (008.47:0) |
|  |  | Intra-abdo abscess postop (998.59:14) |
|  |  | Intra-abdominal sepsis (038.9:2) |
|  |  | Intraabdominal abscess (567.2:4) |
|  |  | Intraperitoneal Abscess (567.2:5) |
|  |  | Ischio-rectal abscess, bilateral (566:1) |
|  |  | Ischiorectal Abscess (566:5) |
|  |  | Leakage fr pancreatic tail (997.4:17) |
|  |  | Leakage from choledochotomy (997.4:16) |
|  |  | Leakage from gastrostomy (997.4:31) |
|  |  | Leakage in GI tract postop (997.4:33) |
|  |  | Leakage of bile, post-operative (997.4:10001) |
|  |  | Leaking accessory bile duct (997.4:19) |
|  |  | Leaking duodenal stump (997.4:21) |
|  |  | Leaking duodenostomy (997.4:22) |
|  |  | Leaking from gallbladder bed (997.4:23) |
|  |  | Leaking GI anastomosis, postop (997.4:20) |
|  |  | Localized salmonella infection (003.20:0) |
|  |  | Necrotizing pancreatitis (577.0:4) |
|  |  | Neutropenic typhlitis (540.9:5) |
|  |  | Oeso herpes simplex infection (054.79:2) |
|  |  | Open drain liver abscess (50.0:5) |
|  |  | Paracolic Abscess (567.2:7) |
|  |  | Parasitology, lower GI (90.94:0) |
|  |  | Paratyphoid fever (002.9:0) |
|  |  | Paratyphoid fever A (002.1:0) |
|  |  | Paratyphoid fever B (002.2:0) |
|  |  | Paratyphoid fever C (002.3:0) |
|  |  | Perforated gallbladder (575.4:0) |
|  |  | Perforated Jejunum (569.83:4) |
|  |  | Perforated rectum (569.49:5) |
|  |  | Perforated sigmoid colon (569.83:7) |
|  |  | Perforated small & large bowel (569.83:8) |
|  |  | Perforated terminal ileum (569.83:10) |
|  |  | Perforated transverse colon (569.83:11) |
|  |  | Perforation esophagus & bleeding (530.4:2) |
|  |  | Perforation GI tract (569.83:13) |
|  |  | Perforation of bile duct (576.3:0) |
|  |  | Perforation of duodenum (569.83:1) |
|  |  | Perforation of esophagus (530.4:0) |
|  |  | Perforation of intestine (569.83:0) |
|  |  | Perforation of proximal jejunum (569.83:5) |
|  |  | Perforation of rectosigmoid colon (569.83:6) |
|  |  | Perforation retroperitoneal organ (568.89:5) |
|  |  | Peritonitis (567.9:0) |
|  |  | Peritonitis, primary (567.9:2) |
|  |  | Peritonitis, Suppurative (567.2:0) |
|  |  | Plesio shigelloide bowel infection (008.47:1) |
|  |  | Pneumococcal peritonitis (567.1:0) |
|  |  | Pneumoperitoneum (568.89:3) |
|  |  | Proteus enteritis (008.3:0) |
|  |  | Proteus species infection (041.6:5) |
|  |  | Protozoal intestinal disease (007.9:0) |
|  |  | Pseudomonas intestinal infection (008.42:0) |
|  |  | Rectal perforation with bleeding (569.49:9) |
|  |  | Repair of perforation, small bowel (46.79:1) |
|  |  | Retroperitoneal Abscess (567.2:8) |
|  |  | Retroperitoneal abscess drainage (54.0:2) |
|  |  | Rupture Esophagus (530.4:1) |
|  |  | Salmonella GE (003.0:0) |
|  |  | Salmonella infection (003.9:0) |
|  |  | Sclerosing peritonitis (567.8:4) |
|  |  | Sclerosing peritonitis, dialysis (567.8:5) |
|  |  | Shigella dysenteriae (004.0:1) |
|  |  | Shigella dysentery (004.0:0) |
|  |  | Shigellosis (004.9:0) |
|  |  | Shigellosis by shigella flexneri (004.1:0) |
|  |  | Shigellosis by shigella sonnei (004.3:0) |
|  |  | Small bowel perforation (569.83:9) |
|  |  | Spontaneous bacterial peritonitis (567.2:9) |
|  |  | Subhepatic Abscess (567.2:2) |
|  |  | Subphrenic abscess (567.2:3) |
|  |  | TB abdomen (014.80:2) |
|  |  | TB adrenal gland - no exam (017.61:0) |
|  |  | TB adrenal gland (017.60:0) |
|  |  | TB bowel+ mesen gland- no exam (014.81:0) |
|  |  | TB bowel+ mesent gland by culture (014.84:0) |
|  |  | TB bowel+ mesent gland by histo (014.85:0) |
|  |  | TB bowel+ mesent gland by micro (014.83:0) |
|  |  | TB bowel+ mesent gland- exam unkno (014.82:0) |
|  |  | TB bowel+ mesenter gland confirmed (014.86:0) |
|  |  | TB esophagus (017.80:0) |
|  |  | TB esophagus by histology (017.85:0) |
|  |  | TB esophagus confirmed (017.86:0) |
|  |  | TB ileitis (014.80:0) |
|  |  | TB peritonitis - exam unknown (014.02:0) |
|  |  | TB peritonitis - no exam (014.01:0) |
|  |  | TB peritonitis (014.00:0) |
|  |  | TB peritonitis by culture (014.04:0) |
|  |  | TB peritonitis by histo (014.05:0) |
|  |  | TB peritonitis by micro exam (014.03:0) |
|  |  | TB peritonitis confirmed (014.06:0) |
|  |  | TB spleen (017.70:0) |
|  |  | Viral gastritis (535.40:3) |
|  |  | Viral gastroenteritis (008.8:0) |
|  |  | Viral hep C carrier (V02.62:0) |
|  |  | Viral hepatitis (070.59:0) |
|  |  | Viral hepatitis (070.9:0) |
|  |  | Viral hepatitis A (070.1:0) |
|  |  | Viral hepatitis C (070.51:1) |
|  | | |
| **Musculoskeletal** | Joint fluid | Abras forearm, elbow, wrist infect (913.1:0) |
|  | Bone | Abscess of bursa (727.89:6) |
|  | Knee joint fluid, Left | Abscess of tendon sheath (727.89:2) |
|  | Knee joint fluid, Right | Ac osteomyelitis lower leg (730.06:0) |
|  | Knee joint fluid | Ac osteomyelitis-ankle (730.07:0) |
|  | Synovium | Ac osteomyelitis-forearm (730.03:0) |
|  | Knee aspirate | Ac osteomyelitis-hand (730.04:0) |
|  | Synovial fluid | Ac osteomyelitis-mult (730.09:0) |
|  | Harvested bone | Ac osteomyelitis-pelvis (730.05:0) |
|  |  | Ac osteomyelitis-up/arm (730.02:0) |
|  |  | Acute hema osteomyel pelvis& thigh (730.05:3) |
|  |  | Acute hema osteomyelit ankle& foot (730.07:3) |
|  |  | Acute hema osteomyelitis lower leg (730.06:2) |
|  |  | Acute hematog osteomyelitis hand (730.04:1) |
|  |  | Acute hematogenous osteomyelitis (730.00:3) |
|  |  | Acute infective arthritis (711.90:3) |
|  |  | Acute osteomyelitis (730.00:0) |
|  |  | Acute osteomyelitis of ankle (730.07:2) |
|  |  | Acute osteomyelitis of foot (730.07:1) |
|  |  | Acute osteomyelitis, shoulder (730.01:0) |
|  |  | Acute suppurat bacterial arthritis (711.00:10) |
|  |  | Arthritis, bacterial infection (711.00:4) |
|  |  | Arthritis, staphylococcal infect (711.00:2) |
|  |  | Arthritis, streptococcal infection (711.00:3) |
|  |  | Arthrotomy & drainage knee (80.16:1) |
|  |  | Bact arthritis-ankle (711.47:0) |
|  |  | Bact arthritis-forearm (711.43:0) |
|  |  | Bact arthritis-hand (711.44:0) |
|  |  | Bact arthritis-l/leg (711.46:0) |
|  |  | Bact arthritis-pelvis (711.45:0) |
|  |  | Bact arthritis-shlder (711.41:0) |
|  |  | Bacterial arthritis upper arm (711.42:0) |
|  |  | Blister finger - infected (915.3:0) |
|  |  | Blister forearm,elbow,wrist infect (913.3:0) |
|  |  | Blister hand-infected (914.3:0) |
|  |  | Blister hip,leg,thigh,ankle-infect (916.3:0) |
|  |  | Blister, infected (919.3:0) |
|  |  | Bone infect - ankle (730.97:0) |
|  |  | Bone infect (730.80:0) |
|  |  | Bone infect (730.88:0) |
|  |  | Bone infect ankle (730.87:0) |
|  |  | Bone infect hand (730.84:0) |
|  |  | Bone infect l/leg (730.86:0) |
|  |  | Bone infect pelvis (730.85:0) |
|  |  | Bone infect shlder (730.81:0) |
|  |  | Bone infect up/arm (730.82:0) |
|  |  | Bone infection - hand (730.94:0) |
|  |  | Bone infection - pelvis (730.95:0) |
|  |  | Bone infection - shoulder (730.91:0) |
|  |  | Bone infection (730.90:0) |
|  |  | Bone infection forearm (730.93:0) |
|  |  | Bone infection, lower leg (730.96:0) |
|  |  | Bone infection, up arm (730.92:0) |
|  |  | Cellulitis & abscess of leg (682.6:0) |
|  |  | Cellulitis/abscess foot, not toes (682.7:0) |
|  |  | Chr osteomyeli w sinus ankle& foot (730.17:4) |
|  |  | Chr osteomyeli w sinus pelv& thigh (730.15:2) |
|  |  | Chr osteomyelit-ankle (730.17:0) |
|  |  | Chr osteomyelit-forearm (730.13:0) |
|  |  | Chr osteomyelit-hand (730.14:0) |
|  |  | Chr osteomyelit-pelvis (730.15:0) |
|  |  | Chr osteomyelit-up/arm (730.12:0) |
|  |  | Chr osteomyelitis, shoulder (730.11:0) |
|  |  | Chro hem osteomyelitis ankle& foot (730.17:5) |
|  |  | Chro osteomyeli w sinus multi site (730.19:2) |
|  |  | Chro osteomyelit w sinus lower leg (730.16:2) |
|  |  | Chro osteomyelit w sinus upper arm (730.12:2) |
|  |  | Chro osteomyeliti w sinus shoulder (730.11:2) |
|  |  | Chron hematogen osteomyelitis hand (730.14:3) |
|  |  | Chron osteomyelitis lower leg (730.16:0) |
|  |  | Chronic infective arthritis (711.90:5) |
|  |  | Chronic multifocal osteomyelitis (730.10:2) |
|  |  | Chronic osteomyelitis (730.10:0) |
|  |  | Chronic osteomyelitis of ankle (730.17:2) |
|  |  | Chronic osteomyelitis of foot (730.17:1) |
|  |  | Chronic osteomyelitis w sinus (730.10:4) |
|  |  | Chronic osteomyelitis w sinus hand (730.14:2) |
|  |  | Disarticulation of hip (84.18:0) |
|  |  | Excis debridement wound under LA (86.22:18) |
|  |  | Extensive fasciitis (729.4:1) |
|  |  | Fasciitis (729.4:0) |
|  |  | FB forearm, infected (913.7:0) |
|  |  | FB head, infected (910.7:0) |
|  |  | FB hip/ leg infected (916.7:0) |
|  |  | FB shoulder/arm, infected (912.7:0) |
|  |  | Foreign body foot+ toe infected (917.7:0) |
|  |  | Foreign body in hand, infected (914.7:0) |
|  |  | Foreign body in hand, infected (914.7:1) |
|  |  | Gonococc synovitis, tenosynovitis (098.51:0) |
|  |  | Gonococcal arthritis (098.50:0) |
|  |  | Gonococcal spondylitis (098.53:0) |
|  |  | I & D hand infection (82.04:1) |
|  |  | I & D palmar / thenar space (82.04:0) |
|  |  | I&D cranial sinus (01.21:0) |
|  |  | I&D deep abscess, hand (82.02:1) |
|  |  | I&D deep abscess, not hand (83.02:2) |
|  |  | I&D shin abscess (86.04:4) |
|  |  | I&D skin / subcutaneous tissue (86.04:999) |
|  |  | Inf arthrit of pelvis (711.95:0) |
|  |  | Inf arthrit oth site (711.98:0) |
|  |  | Inf arthritis ankle (711.87:0) |
|  |  | Inf arthritis hand (711.84:0) |
|  |  | Inf arthritis lower leg (711.86:0) |
|  |  | Inf arthritis of upper arm (711.92:0) |
|  |  | Infect adhesiv capsulitis shoulder (726.0:3) |
|  |  | Infect cervical paravertebral spac (728.0:7) |
|  |  | Infected amputated stump (997.62:0) |
|  |  | Infected joint prosthesis (996.66:0) |
|  |  | Infection of lumbar muscle (728.0:3) |
|  |  | Infection of paravertebral muscle (728.0:4) |
|  |  | Infection orthopaedic device (996.67:2) |
|  |  | Infective arthritis (711.90:0) |
|  |  | Infective arthritis lower leg (711.96:0) |
|  |  | Infective arthritis of ankle (711.97:0) |
|  |  | Infective arthritis of hand (711.94:0) |
|  |  | Infective arthritis shoulder (711.91:0) |
|  |  | Infective bursitis (727.3:2) |
|  |  | Infective myositis (728.0:0) |
|  |  | Infective spondylodiscitis (722.90:5) |
|  |  | Infective spondylopathy (730.98:2) |
|  |  | Infective synovitis (727.09:5) |
|  |  | Infective tenosynovitis (727.09:6) |
|  |  | Intra-spinal abscess, epidural (324.1:3) |
|  |  | Intramuscular abscess (728.89:1) |
|  |  | Major joint infection (711.90:1) |
|  |  | Musculoskeletal system infection (781.9:12) |
|  |  | Mycotic arthritis - ankle (711.67:0) |
|  |  | Necrotizing fasciitis (728.86:0) |
|  |  | Non-TB septic spondylitis (720.9:1) |
|  |  | Non-TB spinal cord abscess (324.1:1) |
|  |  | Nonexcise debridement wound (86.28:0) |
|  |  | Osteomyelitis - ankle (730.27:0) |
|  |  | Osteomyelitis - shoulder (730.21:0) |
|  |  | Osteomyelitis (730.20:0) |
|  |  | Osteomyelitis cervical spine (730.28:6) |
|  |  | Osteomyelitis Femur (730.05:1) |
|  |  | Osteomyelitis lumbar spine (730.28:10) |
|  |  | Osteomyelitis multi sites (730.29:0) |
|  |  | Osteomyelitis occi-atla-axial (730.28:5) |
|  |  | Osteomyelitis of jaw (526.4:2) |
|  |  | Osteomyelitis of pelvis (730.05:2) |
|  |  | Osteomyelitis of sacrum (730.28:1) |
|  |  | Osteomyelitis of vertebral body (730.28:2) |
|  |  | Osteomyelitis pelvis+ thigh (730.25:0) |
|  |  | Osteomyelitis sacrococcygeal spine (730.28:12) |
|  |  | Osteomyelitis spine acute (730.08:1) |
|  |  | Osteomyelitis thoracic spine (730.28:8) |
|  |  | Osteomyelitis thoracolumbar spine (730.28:9) |
|  |  | Osteomyelitis Tibia (730.06:1) |
|  |  | Osteomyelitis upper arm (730.22:0) |
|  |  | Osteomyelitis, forearm (730.23:0) |
|  |  | Osteomyelitis, hand (730.24:0) |
|  |  | Osteomyelitis, lower leg (730.26:0) |
|  |  | Pneumococcal arthritis knee (711.06:3) |
|  |  | Post-op wound infection (998.59:3) |
|  |  | Pyogenic arthritis (711.00:0) |
|  |  | Pyogenic arthritis, ankle & foot (711.07:0) |
|  |  | Pyogenic arthritis, elbow (711.02:1) |
|  |  | Pyogenic arthritis, hand (711.04:0) |
|  |  | Pyogenic arthritis, hip (711.05:1) |
|  |  | Pyogenic arthritis, knee (711.06:1) |
|  |  | Pyogenic arthritis, multiple sites (711.09:0) |
|  |  | Pyogenic arthritis, pelvis & thigh (711.05:0) |
|  |  | Pyogenic arthritis, shoulder (711.01:0) |
|  |  | Pyogenic arthritis, wrist (711.03:1) |
|  |  | Remove implant device from femur (78.65:0) |
|  |  | Salmonella arthritis (003.23:0) |
|  |  | Salmonella osteomyelitis (003.24:0) |
|  |  | Septic arthritis atlantoaxial jt (711.08:1) |
|  |  | Septic arthritis cervical facet jt (711.08:2) |
|  |  | Septic arthritis costotransvers jt (711.08:4) |
|  |  | Septic arthritis costovertebral jt (711.08:5) |
|  |  | Septic arthritis lumbar facet jt (711.08:3) |
|  |  | Septic arthritis sacroiliac joint (711.05:2) |
|  |  | Septic arthritis thoracic facet jt (711.08:6) |
|  |  | Sequestrectom infected facial bone (76.01:2) |
|  |  | Spinal abscess (324.1:2) |
|  |  | Staph arthritis ankle (711.07:6) |
|  |  | Staph arthritis elbow (711.02:2) |
|  |  | Staph arthritis hip (711.05:3) |
|  |  | Staph arthritis knee (711.06:2) |
|  |  | Staph arthritis multi sites (711.09:1) |
|  |  | Strepto arthritis knee (711.06:4) |
|  |  | Strepto arthritis wrist (711.03:4) |
|  |  | Streptococcus suis arthritis (711.00:5) |
|  |  | Subacute infective arthritis (711.90:7) |
|  |  | Subacute osteomyelit pelvis& thigh (730.05:4) |
|  |  | Subacute osteomyelitis (730.00:5) |
|  |  | Subacute osteomyelitis ankle& foot (730.07:4) |
|  |  | Subacute osteomyelitis forearm (730.03:2) |
|  |  | Subacute osteomyelitis hand (730.04:2) |
|  |  | Subacute osteomyelitis lower leg (730.06:3) |
|  |  | Subacute osteomyelitis shoulder (730.01:2) |
|  |  | Subacute osteomyelitis upper arm (730.02:2) |
|  |  | TB arthritis ankle & foot (015.90:3) |
|  |  | TB arthritis elbow (015.80:5) |
|  |  | TB arthritis forearm (015.80:3) |
|  |  | TB arthritis hand (015.80:4) |
|  |  | TB arthritis lower leg (015.20:1) |
|  |  | TB arthritis pelvis & thigh (015.10:1) |
|  |  | TB arthritis shoulder (015.80:1) |
|  |  | TB arthritis upper arm (015.80:2) |
|  |  | TB bone - exam unknown (015.72:0) |
|  |  | TB bone - no exam (015.71:0) |
|  |  | TB bone + joint by micro exam (015.93:0) |
|  |  | TB bone + joint- exam unknown (015.92:0) |
|  |  | TB bone and joints (015.90:0) |
|  |  | TB bone by culture (015.74:0) |
|  |  | TB bone by histology (015.75:0) |
|  |  | TB bone by micro exam (015.73:0) |
|  |  | TB bone confirmed (015.76:0) |
|  |  | TB bone+ joint by culture (015.94:0) |
|  |  | TB bones and joints, confirmed (015.96:0) |
|  |  | TB cervical neck abscess (017.20:1) |
|  |  | TB hip - exam unknown (015.12:0) |
|  |  | TB hip - no exam (015.11:0) |
|  |  | TB hip (015.10:0) |
|  |  | TB hip by culture (015.14:0) |
|  |  | TB hip by histology (015.15:0) |
|  |  | TB hip by micro exam (015.13:0) |
|  |  | TB hip confirmed (015.16:0) |
|  |  | TB joint (015.80:0) |
|  |  | TB joint by culture (015.84:0) |
|  |  | TB joint by histology (015.85:0) |
|  |  | TB joint by micro exam (015.83:0) |
|  |  | TB joint confirmed (015.86:0) |
|  |  | TB knee - no exam (015.21:0) |
|  |  | TB knee by culture (015.24:0) |
|  |  | TB knee by histology (015.25:0) |
|  |  | TB knee by micro exam (015.23:0) |
|  |  | TB knee confirmed (015.26:0) |
|  |  | TB kyphosis (015.00:2) |
|  |  | TB limb bones - no exam (015.51:0) |
|  |  | TB limb bones by culture (015.54:0) |
|  |  | TB limb bones by histology (015.55:0) |
|  |  | TB limb bones by micro exam (015.53:0) |
|  |  | TB limb bones confirmed (015.56:0) |
|  |  | TB neck abscess (017.00:0) |
|  |  | TB of knee (015.20:0) |
|  |  | TB of limb bones (015.50:0) |
|  |  | TB spinal cord (013.40:1) |
|  |  | TB spinal cord abscess by culture (013.54:0) |
|  |  | TB spinal cord abscess by histo (013.55:0) |
|  |  | TB spinal cord abscess- no exam (013.51:0) |
|  |  | TB spine (015.00:0) |
|  |  | TB spondylitis (015.00:3) |
|  |  | TB vertebra - no exam (015.01:0) |
|  |  | TB vertebra by culture (015.04:0) |
|  |  | TB vertebra by micro exam (015.03:0) |
|  |  | TB vertebra confirmed (015.06:0) |
|  |  | TB vertebra- exam unknown (015.02:0) |
|  |  | Tuberculosis bone in lower leg (015.50:4) |
|  |  | Tuberculosis bone in upper arm (015.50:5) |
|  |  | Tuberculosis bone of ankle & foot (015.50:2) |
|  |  | Tuberculosis bone pelvis & thigh (015.50:3) |
|  |  | Tuberculosis bone shoulder region (015.50:7) |
|  |  | Tuberculosis of bone (015.70:0) |
|  |  | Tuberculosis of bone (015.90:6) |
|  |  | Tuberculosis of hand bone (015.50:1) |
|  |  | Tuberculosis with arthritis (015.90:1) |
|  |  | Unilateral IH with gangrene (550.00:0) |
|  |  | Viral arthritis - multiple (711.59:0) |
|  |  | Viral arthritis upper arm (711.52:0) |
|  |  | Viral arthritis-ankle (711.57:0) |
|  |  | Viral arthritis-hand (711.54:0) |
|  | | |
| **Neurological** | Cerebrospinal fluid | Acute meningococcemia (036.2:1) |
|  | External ventricular drain tip | Anaerobic meningitis (320.81:0) |
|  | Ventricular catheter | Asymptomatic neurosyphilis (094.3:0) |
|  | Subdural fluid | Bacterial meningitis (320.9:0) |
|  | Ventricular tap | Bacterial meningitis, post-op (320.9:1) |
|  | Brain abscess | Bacterial meningoencephalitis (320.9:3) |
|  | Epidural fluid | Brain abscess (324.0:1) |
|  | Ventriculoperitoneal shunt | Burr hole drain subdural empyema (01.31:4) |
|  | Catheter, brain related | Candidal meningitis (112.83:0) |
|  | Ventricular catheter tip | Central nervous system infection (349.89:4) |
|  | Brain tissue | Chronic meningitis (322.2:0) |
|  | Pendenz valve | CNS TB - exam unknown (013.92:0) |
|  | Ventriculoperitoneal shunt, cerebrospinal fluid | CNS TB - no exam (013.91:0) |
|  |  | CNS TB by culture (013.94:0) |
|  |  | CNS TB by micro exam (013.93:0) |
|  |  | CNS TB confirmed (013.96:0) |
|  |  | Cryptococcal meningitis (117.5:1) |
|  |  | Cryptococcal meningitis (321.0:0) |
|  |  | Cryptococcosis (117.5:0) |
|  |  | Cryptococcosis asso with AIDS (042:49) |
|  |  | Encephalitis (323.8:0) |
|  |  | Encephalitis (323.9:0) |
|  |  | Encephalitis due to infection (323.4:0) |
|  |  | Encephalitis in viral disease (323.0:0) |
|  |  | Encephalomeningocele (742.0:7) |
|  |  | Encephalomyelitis (323.9:1) |
|  |  | Fungal meningitis (117.9:3) |
|  |  | Fungal meningitis (321.1:0) |
|  |  | Gram-negative meningitis (320.82:0) |
|  |  | Hemophilus meningitis (320.0:0) |
|  |  | Herpes simplex encephalitis (054.3:1) |
|  |  | Herpes simplex meningitis (054.72:0) |
|  |  | Herpes zoster meningitis (053.0:0) |
|  |  | Herpetic meningoencephalitis (054.3:0) |
|  |  | HIV-2 causing CNS disorder (042:21) |
|  |  | I&D of brain abscess (01.39:1) |
|  |  | Infection of epidural space (324.1:6) |
|  |  | Infection of intervertebral disc (722.90:3) |
|  |  | Infection of intervertebral disk (722.90:2) |
|  |  | Infection of neurological system (136.9:7) |
|  |  | Infective meningitis (320.9:2) |
|  |  | Intracranial & intraspinal abscess (324.9:0) |
|  |  | Intracranial abscess (324.0:0) |
|  |  | Intracranial abscess, extradural (324.0:5) |
|  |  | Intracranial abscess, subdural (324.0:6) |
|  |  | Intraspinal abscess (324.1:0) |
|  |  | Japanese encephalitis (062.0:0) |
|  |  | Listerial sepsis (027.0:4) |
|  |  | Listerial septicemia (027.0:5) |
|  |  | Lyme disease (088.81:0) |
|  |  | Meningitis (322.9:0) |
|  |  | Meningitis due to virus (321.2:0) |
|  |  | Meningitis in bacterial disease (320.7:0) |
|  |  | Meningitis, post-op (322.9:2) |
|  |  | Meningococcal encephalitis (036.1:0) |
|  |  | Meningococcal infection (036.9:0) |
|  |  | Meningococcal meningitis (036.0:0) |
|  |  | Meningococcemia (036.2:0) |
|  |  | Meningoencephalitis (323.9:10) |
|  |  | Meningovascular syphilis, cerebral (094.2:1) |
|  |  | Myelomeningocele, spinal (741.90:3) |
|  |  | Neurosyphilis (094.9:0) |
|  |  | Non-TB brain abscess (324.0:2) |
|  |  | Occipital meningocele (742.0:3) |
|  |  | Open Bx cerebral meninges (01.12:0) |
|  |  | Pneumococcal meningitis (320.1:0) |
|  |  | Salmonella meningitis (003.21:0) |
|  |  | Slow virus infection CNS (046.9:0) |
|  |  | Staphylococcal meningitis (320.3:0) |
|  |  | Streptococcal meningitis (320.2:0) |
|  |  | Streptococcus suis meningitis (320.2:1) |
|  |  | Subarachnoid abscess (324.0:4) |
|  |  | TB abscess of brain (013.30:0) |
|  |  | TB abscess of spinal cord (013.50:0) |
|  |  | TB arachnoiditis (013.00:1) |
|  |  | TB brain abscess - no exam (013.31:0) |
|  |  | TB brain abscess exam unknown (013.32:0) |
|  |  | TB encephalitis/ myelitis (013.60:0) |
|  |  | TB epidural abscess (013.80:2) |
|  |  | TB lesion in brain (013.20:0) |
|  |  | TB meningitis - exam unknown (013.02:0) |
|  |  | TB meningitis (013.00:0) |
|  |  | TB meningitis by culture (013.04:0) |
|  |  | TB meningitis by histology (013.05:0) |
|  |  | TB meningitis by microscopic exam (013.03:0) |
|  |  | TB meningitis confirmed (013.06:0) |
|  |  | TB myelitis (013.60:1) |
|  |  | TB spinal cord abscess confirmed (013.56:0) |
|  |  | Tuberculoma brain - exam unknown (013.22:0) |
|  |  | Tuberculoma brain - no exam (013.21:0) |
|  |  | Tuberculoma brain by histology (013.25:0) |
|  |  | Tuberculoma of meninges (013.10:0) |
|  |  | Tuberculoma spinal cord (013.40:0) |
|  |  | Viral encephalitis (049.8:1) |
|  |  | Viral meningitis (047.9:0) |
|  |  | Zoster encephalitis (053.19:2) |
|  | | |
| **Cardiac** | Pericardial fluid | Ac endocardit in oth dis (421.1:0) |
|  |  | Acute bacterial endocarditis (421.0:1) |
|  |  | Acute endocarditis (421.9:0) |
|  |  | Candidal endocarditis (112.81:0) |
|  |  | Coxsackie myocarditis (074.23:0) |
|  |  | Coxsackie virus infection (079.2:0) |
|  |  | Endocarditis (424.90:0) |
|  |  | Endocarditis in other disease (424.91:0) |
|  |  | Endocarditis, bacterial (421.0:2) |
|  |  | Gonococcal endocarditis (098.84:0) |
|  |  | Infect endocarditis by card shunt (421.0:4) |
|  |  | Infective endocarditis (421.0:999) |
|  |  | Infective pericarditis (420.90:1) |
|  |  | Meningococc myocarditis (036.43:0) |
|  |  | Mycotic endocarditis (421.0:6) |
|  |  | Pericarditis with infective cause (420.90:2) |
|  |  | Septic myocarditis (422.92:0) |
|  |  | Streptococcus suis endocarditis (421.0:5) |
|  |  | TB pericarditis (017.90:2) |
|  |  | Viral myocarditis (422.91:2) |
|  | | |
| **Eye/Dental/ENT** | Throat swab | Abscess of hard palate (526.4:4) |
|  | Corneal scraping, Left | Abscess of Nasal Septum (478.1:1) |
|  | Eye swab, Left | Abscess of nasopharynx (478.29:3) |
|  | Eye swab, Right | Abscess of oral soft tissue (528.3:8) |
|  | Eye swab | Abscess of pharynx (478.29:2) |
|  | Ear swab | Ac epiglottitis w obstr (464.31:0) |
|  | Corneal scraping, Right | Acute apical abscess (522.5:2) |
|  | Ear swab, Left | Acute apical periodontitis (522.4:0) |
|  | Tongue swab | Acute canaliculitis (375.31:0) |
|  | Ear swab, Right | Acute dacryoadenitis (375.01:0) |
|  | Vitreous tap, Left | Acute dacryocystitis (375.32:0) |
|  | Conjunctival scraping, Left | Acute endophthalmitis (360.01:0) |
|  | Oral swab | Acute epiglottitis (464.30:0) |
|  | Conjunctival swab, Right | Acute mucoid otitis media (381.02:0) |
|  | Vitreous tap, Right | Acute myringitis (384.00:0) |
|  | Conjunctival swab | Acute nasopharyngitis (460:0) |
|  | Corneal scraping | Apical periodontitis (522.6:3) |
|  | Tonsil swab | Candidal otitis externa (112.82:0) |
|  | Contact lens, Left | Candidiasis mouth asso w AIDS (042:46) |
|  | Contact lens, Right | Candidiasis of esophagus (112.84:0) |
|  | Contact lens | Cellulitis/abscess mouth (528.3:0) |
|  | Vitreous tap | Chronic canaliculitis (375.41:0) |
|  | Conjunctival swab, Left | Chronic infective otitis externa (380.16:0) |
|  | Ocular swab | Corneal abscess (370.55:0) |
|  | Ocular fluid | Dental abscess (522.5:1) |
|  | Dental swab | Drain dental abscess (24.99:13) |
|  | Donor corneal storage medium, Right | Drain face & mouth floor (27.0:0) |
|  | Donor corneal rim, Right | Follicular tonsillitis (463:3) |
|  | Gum swab | Herpes zoster iridocyclitis (053.22:0) |
|  | Ear discharge, Left | Herpes zoster keratoconjunctivitis (053.21:0) |
|  | Ear discharge, Right | Herpes zoster ophthalmicus (053.20:1) |
|  | Ear discharge | Herpes zoster otitis externa (053.71:0) |
|  | Donor corneal rim | Herpes zoster w eye complication (053.29:0) |
|  | Donor corneal storage medium | I & D of lacrimal passage (09.59:1) |
|  | Conjunctival scraping, Right | I&D of peritonsillar abscess (28.0:4) |
|  | Donor corneal rim, Left | I&D preauricular abscess (86.04:11) |
|  | Donor corneal storage medium, Left | I&D retropharyng abscess ext appro (28.0:2) |
|  | Corneal swab | I&D retropharyng abscess intraoral (28.0:3) |
|  |  | I&D, parapharyngeal abscess (28.0:1) |
|  |  | Infection of eye (360.00:2) |
|  |  | Infectious conjunctivitis (372.30:1) |
|  |  | Infective dermatitis of eyelid (373.5:0) |
|  |  | Mastoiditis (383.9:0) |
|  |  | Mastoiditis, Acute (383.00:0) |
|  |  | Mycotic corneal ulcer (370.05:0) |
|  |  | Nasopharyngitis (460:1) |
|  |  | Orbital cellulitis (376.01:0) |
|  |  | Otitis media (382.9:0) |
|  |  | Parapharyngeal abscess (478.22:0) |
|  |  | Parasitic conjunctivitis (372.15:0) |
|  |  | Periapical abscess with sinus (522.7:0) |
|  |  | Periapical abscess without sinus (522.5:0) |
|  |  | Periapical periodontitis (522.6:1) |
|  |  | Periodontal abscess (523.3:1) |
|  |  | Postseptal orbital cellulitis (376.01:3) |
|  |  | Retropharyngeal abscess (478.24:0) |
|  |  | Salivary gland abscess (527.3:0) |
|  |  | Secondary iritis, infectious (364.03:0) |
|  |  | Strep sore throat (034.0:0) |
|  |  | Streptococcal tonsillitis (034.0:1) |
|  |  | Submandibular gland infection (527.8:1) |
|  |  | Submucosal abscess, oral cavity (528.3:3) |
|  |  | Subperi mastoid abscess (383.01:0) |
|  |  | Suppurative otitis media (382.4:0) |
|  |  | TB disseminated chorioretinitis (017.30:1) |
|  |  | TB ear (017.40:0) |
|  |  | TB ear by culture (017.44:0) |
|  |  | TB episcleritis (017.30:2) |
|  |  | TB eye - exam unknown (017.32:0) |
|  |  | TB eye (017.30:0) |
|  |  | TB laryngitis by histology (012.35:0) |
|  |  | TB larynx (012.30:1) |
|  |  | TB mastoid confirmed (015.66:0) |
|  |  | TB nasopharynx / oropharynx (012.80:2) |
|  |  | TB thyroid gland (017.50:0) |
|  |  | Trichiasis of eyelid (374.05:0) |
|  |  | Tuberculous laryngitis (012.30:0) |
|  |  | Viral conjunctivitis (077.8:0) |
|  |  | Viral labyrinthitis (386.35:0) |
|  | | |
| **Prothesis** | Catheter non IV | Dialysis exit site infection (998.59:12) |
|  | Catheter IV | Gastrostomy site infection (996.69:3) |
|  | Catheter tip | Infec due to hrt device (996.61:0) |
|  | Tenckhoff catheter exit site swab | Infec due to nerv device (996.63:0) |
|  | Catheter IV, CVP | Infec due to vasc device (996.62:0) |
|  | Hickman swab | Infect by implanted cardiac device (996.61:3) |
|  | Catheter tip IV, CVP | Infect filtering bleb postop (996.69:2) |
|  | Catheter | Infect/ inflam by device/ graft (996.60:0) |
|  | Catheter IV, Hickman | Infect/ inflam by indwel urin cath (996.64:0) |
|  | Catheter IV, peripheral | Infect/ inflam by int orth device (996.67:0) |
|  | Internal jugular catheter, Right | Infect/ inflam by internal device (996.69:0) |
|  | Tenckhoff catheter tip | Infect+ inflam retained IUCD (996.65:3) |
|  | Subclavian catheter tip, Right | Infected arteriovenous graft (996.62:1) |
|  | Catheter tip IV, peripheral | Infected implants (996.60:1) |
|  | Arterial line tip | Infected VP shunt (996.63:2) |
|  | Subclavian catheter tip, Left | Infection d/t gu device/graft (996.65:0) |
|  | Internal jugular catheter, Left | Infection due to IUCD (996.65:2) |
|  | Catheter tip, umbilical | Infection of breast implant (996.69:5) |
|  | Catheter IV, umbilical artery | Infection of generator site (996.61:1) |
|  | Arterial line | Infection of interbody graft (996.67:6) |
|  |  | Infection of vascular entry site (998.59:15) |
|  |  | Shunt infection, ventricular (996.63:1) |
|  | | |
| **Skin** | Superficial wound swab | Abrasion finger infected (915.1:0) |
|  | Bed sore swab | Abrasion foot/toe infect (917.1:0) |
|  | Skin scraping | Abrasion hand infected (914.1:0) |
|  | Skin swab | Abrasion infected (919.1:0) |
|  | Ulcer swab | Abrasion shoulder/ arm infect (912.1:0) |
|  | Nail | Abrasion shoulder/ upp arm infect (912.1:1) |
|  | Blister fluid | Abrasion trunk infected (911.1:0) |
|  | Nail scraping | Abrasion, infected (919.1:1) |
|  | Skin | Abscess of eyelid (373.13:0) |
|  | Vesicle fluid | Abscess of head, except face (682.8:2) |
|  | Skin biopsy | Abscess of lip (528.5:8) |
|  | Sinus swab | Abscess, upper eyelid (373.13:1) |
|  | Vesicle swab | Acute bacterial paronychia (681.9:3) |
|  | Hair | Acute herpes zoster (053.9:1) |
|  | Axilla swab, Right | Acute infection of external ear (380.13:0) |
|  | Breast aspirate | Acute infection of pinna (380.11:0) |
|  | Breast milk, Left | Acute suppurative otitis media (382.00:0) |
|  | Expressed breast milk | Breast abscess (611.0:1) |
|  | Blister swab | Breast abscess, antepartum care (675.13:800) |
|  | Breast milk, Right | Breast abscess, postpartum care (675.14:800) |
|  | Axilla swab, Left | Candidiasis of skin (112.3:1) |
|  | Milk | Capitis tinea (110.0:1) |
|  |  | Carbuncle (680.9:1) |
|  |  | Carbuncle and furuncle (680.9:0) |
|  |  | Carbuncle arm, forearm (680.3:2) |
|  |  | Carbuncle of abdominal wall (680.2:2) |
|  |  | Carbuncle of arm (680.3:0) |
|  |  | Carbuncle of arm (680.3:1) |
|  |  | Carbuncle of back (680.2:1) |
|  |  | Carbuncle of buttock (680.5:0) |
|  |  | Carbuncle of buttock (680.5:1) |
|  |  | Carbuncle of face (680.0:0) |
|  |  | Carbuncle of face (680.0:1) |
|  |  | Carbuncle of foot (680.7:0) |
|  |  | Carbuncle of foot (680.7:1) |
|  |  | Carbuncle of hand (680.4:0) |
|  |  | Carbuncle of hand (680.4:1) |
|  |  | Carbuncle of leg (680.6:0) |
|  |  | Carbuncle of leg (680.6:1) |
|  |  | Carbuncle of neck (680.1:0) |
|  |  | Carbuncle of neck (680.1:1) |
|  |  | Carbuncle of trunk (680.2:0) |
|  |  | Carbuncle of trunk (680.2:3) |
|  |  | Cellulitis & abscess of toes (681.10:0) |
|  |  | Cellulitis and abscess - arm (682.3:0) |
|  |  | Cellulitis and abscess of ankle (682.6:2) |
|  |  | Cellulitis and abscess of finger (681.00:0) |
|  |  | Cellulitis of buttock (682.5:0) |
|  |  | Cellulitis of external ear (380.10:4) |
|  |  | Cellulitis of face (682.0:0) |
|  |  | Cellulitis of neck (682.1:0) |
|  |  | Cellulitis of pharynx (478.21:0) |
|  |  | Cellulitis of trunk (682.2:5) |
|  |  | Cellulitis pharynx (478.21:1) |
|  |  | Cellulitis site (682.8:0) |
|  |  | Cellulitis, postoperative (682.9:1) |
|  |  | Cellulitis/ abscess finger pulp (681.01:1) |
|  |  | Cellulitis/abscess hand-not finger (682.4:0) |
|  |  | Cutaneous actinomycosis (039.0:0) |
|  |  | Cutaneous candidiasis (112.3:0) |
|  |  | Cutaneous cryptococcosis (117.5:4) |
|  |  | Cutaneous diphtheria (032.85:0) |
|  |  | Cutaneous mycobacterial infection (031.1:0) |
|  |  | Debride wound <5% TBS+GA/sed (86.22:11) |
|  |  | Debride wound 5-<20% TBS+GA/sed (86.22:12) |
|  |  | Exit site infection, post-op (998.59:7) |
|  |  | Exploration of skin wound (86.09:1) |
|  |  | Friction burn, infected (919.1:2) |
|  |  | Furuncle (680.9:2) |
|  |  | Furuncle of back (680.2:5) |
|  |  | Furuncle of buttock (680.5:2) |
|  |  | Furuncle of face (680.0:2) |
|  |  | Furuncle of neck (680.1:2) |
|  |  | Herpes simplex dendritic keratitis (054.42:0) |
|  |  | Herpes simplex dermatitis eyelid (054.41:0) |
|  |  | Herpes simplex disciform keratitis (054.43:0) |
|  |  | Herpes zoster auricularis (053.71:1) |
|  |  | Herpes zoster dermatitis eyelid (053.20:0) |
|  |  | Herpes zoster infection (053.9:0) |
|  |  | I & D of breast abscess (85.0:1) |
|  |  | I&B external ear (18.09:1) |
|  |  | I&D of lesion of abdominal wall (54.0:4) |
|  |  | Infect/ abscess Cesarean wound, PP (674.34:810) |
|  |  | Infected breast+ nipple, postpartu (675.94:800) |
|  |  | Infected finger nail bed (681.02:3) |
|  |  | Infected thyroidectomy wound (998.59:6) |
|  |  | Infection of surgical fusion site (996.67:3) |
|  |  | Infectious mastitis, postpartum (675.14:802) |
|  |  | Insect bite foot / toe, infected (917.5:0) |
|  |  | Insect bite hand-infect (914.5:0) |
|  |  | Insect bite head-infect (910.5:0) |
|  |  | Insect bite infected (919.5:0) |
|  |  | Insect bite shoulder / arm, infect (912.5:0) |
|  |  | Insect bite trunk - infected (911.5:0) |
|  |  | Local skin infection (686.9:0) |
|  |  | Mycosis fungoides, head (202.11:0) |
|  |  | Mycosis fungoides, inguinal (202.15:0) |
|  |  | Otitis externa infective (380.10:0) |
|  |  | Paronychia in finger (681.02:2) |
|  |  | Paronychia in toe (681.11:2) |
|  |  | Periauricular Abscess (380.10:1) |
|  |  | Post-trauma skin infection (686.9:10) |
|  |  | Posttrauma wound infection (958.3:0) |
|  |  | Preauricular sinus infection (380.10:3) |
|  |  | Sacral abscess (730.08:2) |
|  |  | Skin abscess (682.9:10) |
|  |  | Skin infection of finger (686.9:9) |
|  |  | Skin infection of toe (686.9:7) |
|  |  | Subungual abscess (681.9:2) |
|  |  | Superf inj forearm elb wri infect (913.9:0) |
|  |  | Superf inj head infected (910.9:0) |
|  |  | Superf injury foot & toe, infected (917.9:0) |
|  |  | Superfi infect FB hand not finger (914.7:2) |
|  |  | Superfical injury hand infected (914.9:0) |
|  |  | Superfical injury hip/ leg infect (916.9:0) |
|  |  | Superficial FB finger, infected (915.7:0) |
|  |  | Superficial FB forearm infected (913.7:1) |
|  |  | Superficial FB hip/ leg infected (916.7:2) |
|  |  | Superficial FB trunk, infected (911.7:1) |
|  |  | Superficial foreign body, infected (919.7:0) |
|  |  | Superficial injury, infected (919.9:0) |
|  |  | Suprficial inj finger infected (915.9:0) |
|  |  | TB sacral abscess (015.00:4) |
|  |  | TB skin (017.00:1) |
|  |  | TB skin & soft tissue w +ve AFB (017.03:1) |
|  |  | TB skin+ subcut tiss by histology (017.05:0) |
|  |  | TB skin+ subcut tiss- exam unknown (017.02:0) |
|  |  | TB skin+ subcutan by micro exam (017.03:0) |
|  |  | TB skin+ subcutan tiss by culture (017.04:0) |
|  |  | TB skin+ subcutane tiss- no exam (017.01:0) |
|  |  | TB skin+ subcuteous tiss confirmed (017.06:0) |
|  |  | Tinea (110.9:1) |
|  |  | Tinea cruris (110.3:3) |
|  |  | Tinea of body (110.5:1) |
|  |  | Tinea of foot (110.4:1) |
|  |  | Tinea of groin and perianal area (110.3:1) |
|  |  | Tinea of hand (110.2:1) |
|  |  | Tinea of nail (110.1:1) |
|  |  | Tuberculoid leprosy (030.1:0) |
|  |  | Viral wart (078.10:0) |
|  |  | Viral warts (078.19:0) |
|  | | |
| **Systemic** | Blood, culture | Acute HIV infection syndrome (042:39) |
|  | Blood | Acute miliary TB - no exam (018.01:0) |
|  | Lymph node | Acute miliary TB (018.00:0) |
|  | Bone marrow aspirate | Acute miliary TB by culture (018.04:0) |
|  | Lymph node biopsy | Acute miliary TB by histology (018.05:0) |
|  | Blood product, donor blood pack | Acute miliary TB by micro exam (018.03:0) |
|  | Bone marrow | Acute miliary TB exam unknown (018.02:0) |
|  | Blood product, red cells | Acute miliary TB, multi sites (018.00:2) |
|  | Blood product, whole blood | Acute miliary TB, single site (018.00:1) |
|  | Blood product, platelet concentrate | Adenovirus infection (079.0:0) |
|  | Blood product, fresh frozen plasma | Aplastic anemia, parvovirus infect (284.8:5) |
|  | Blood product | Asymptom HIV infection status (V08:0) |
|  | Cervical lymph node | Bartonellosis (088.0:0) |
|  |  | Brucella abortus infection (023.1:0) |
|  |  | Brucella melitensis infection (023.0:0) |
|  |  | Brucellosis (023.9:0) |
|  |  | Candidemia (112.5:2) |
|  |  | CMV Infection (078.5:0) |
|  |  | Delivered with syphilis (647.01:800) |
|  |  | Dengue (061:0) |
|  |  | Dengue hemorrhagic fever (065.4:2) |
|  |  | Disseminated aspergillosis (117.3:5) |
|  |  | Disseminated cancer (199.0:0) |
|  |  | Disseminated candidiasis (112.5:0) |
|  |  | Disseminated carcinoma (199.0:1) |
|  |  | Disseminated cryptococcosis (117.5:6) |
|  |  | Disseminated strongyloidiasis (127.2:4) |
|  |  | Disseminated tuberculosis (018.90:1) |
|  |  | Disseminated zoster (053.79:1) |
|  |  | Early syphilis (091.0:1) |
|  |  | Early syphilis, latent (092.9:0) |
|  |  | Echo virus infection (079.1:0) |
|  |  | EIA test for syphilis +ve (795.79:1) |
|  |  | Epstein Barr virus infection (075:1) |
|  |  | EV 71 infection (079.89:5) |
|  |  | Fungal septicemia (117.9:5) |
|  |  | Fungemia (117.9:9) |
|  |  | Gonococcal infection (098.0:2) |
|  |  | Gonococcal infection (098.89:0) |
|  |  | Gonococcus dis carrier (V02.7:2) |
|  |  | Gonorrhea carrier (V02.7:0) |
|  |  | Gonorrhea for antepartum care (647.13:800) |
|  |  | Gonorrhea for postpartum care (647.14:800) |
|  |  | Gonorrhea, female (098.0:3) |
|  |  | Gonorrhea, male (098.89:2) |
|  |  | Hantavirus infection (078.89:4) |
|  |  | Herpes simplex infection (054.9:0) |
|  |  | Herpes simplex iridocyclitis (054.44:0) |
|  |  | Herpes simplex w eye complication (054.40:0) |
|  |  | Herpes simplex with complication (054.8:0) |
|  |  | Herpes zoster with complication (053.8:0) |
|  |  | Herpesvirus infection (054.9:2) |
|  |  | Herpetic gingivostomatitis (054.2:0) |
|  |  | HIV antibody indeterminate (795.71:1) |
|  |  | HIV antibody positive (V08:1) |
|  |  | HIV diseas result in multi disease (042:37) |
|  |  | HIV disease type 2 (042:18) |
|  |  | HIV disease, antepartum care (647.63:800) |
|  |  | HIV disease, delivered (647.61:1) |
|  |  | HIV enteropathy (042:43) |
|  |  | HIV infection (042:3) |
|  |  | HIV infection / AIDS (042:40) |
|  |  | HIV infection causing CNS disorder (042:16) |
|  |  | HIV infection w immun disease (042:17) |
|  |  | HIV infection with cancer (042:14) |
|  |  | HIV infection with lymphadenopathy (042:15) |
|  |  | HIV result in bacterial infection (042:26) |
|  |  | HIV result in candidiasis (042:29) |
|  |  | HIV result in CMV disease (042:27) |
|  |  | HIV result in multi infections (042:32) |
|  |  | HIV result in mycobacterial infect (042:25) |
|  |  | HIV result in mycosis (042:30) |
|  |  | HIV result in pneumocystosis (042:31) |
|  |  | HIV result in viral infection (042:28) |
|  |  | HIV result in wasting syndrome (042:36) |
|  |  | HIV resuting in Burkitt's lymphoma (042:50) |
|  |  | HIV type I infection (042:47) |
|  |  | HIV with specific infection (042:13) |
|  |  | HIV-2 (079.53:0) |
|  |  | HIV-2 with lymphadenopathy (042:22) |
|  |  | Infectious mononucleosis (075:0) |
|  |  | Malaria (084.6:0) |
|  |  | Measles (055.9:0) |
|  |  | Measles with complication (055.8:0) |
|  |  | Miliary TB - exam unknown (018.92:0) |
|  |  | Miliary TB - no exam (018.91:0) |
|  |  | Miliary TB (018.90:0) |
|  |  | Miliary TB by culture (018.94:0) |
|  |  | Miliary TB by histology (018.95:0) |
|  |  | Miliary TB by micro exam (018.93:0) |
|  |  | Miliary TB confirmed (018.96:0) |
|  |  | Necrotizing lymphadenitis (289.3:8) |
|  |  | Parvovirus B19 infection (079.89:2) |
|  |  | Parvovirus B19 infection, antepart (647.63:803) |
|  |  | Parvovirus infection (079.89:1) |
|  |  | Pneumococcal sepsis (038.2:1) |
|  |  | Pneumococcus infection (041.2:0) |
|  |  | Primary syphilis (091.2:0) |
|  |  | Q fever (083.0:0) |
|  |  | Rickettsial encephalitis (323.1:0) |
|  |  | Rickettsialpox (083.2:0) |
|  |  | Rickettsiosis (083.9:0) |
|  |  | Schistosoma japonicum infect (120.2:0) |
|  |  | Schistosoma mansoni infection (120.1:0) |
|  |  | Schistosomiasis (120.9:0) |
|  |  | Scrub typhus (081.2:0) |
|  |  | Staph aureus sepsis (038.1:5) |
|  |  | Staph aureus septicemia (038.1:2) |
|  |  | Syphili dissemin retinochoroiditis (094.83:0) |
|  |  | Syphilis (097.9:0) |
|  |  | Syphilis carrier (V02.8:3) |
|  |  | Syphilis for antepartum care (647.03:800) |
|  |  | Syphilis synovium, tendon, bursa (095.7:0) |
|  |  | Syphilis, female (097.9:1) |
|  |  | Syphilis, male (097.9:2) |
|  |  | Syphilit aortic valve endocarditis (093.22:0) |
|  |  | Syphilitic aortitis (093.1:0) |
|  |  | Syphilitic encephalitis (094.81:0) |
|  |  | Syphilitic meningitis (094.2:0) |
|  |  | Syphilitic Parkinsonism (094.82:0) |
|  |  | Syphilitic retrobulbar neuritis (094.85:0) |
|  |  | Syphilitic uveitis (091.50:0) |
|  |  | TB cervical lymph node in neck (017.20:2) |
|  |  | TB intrathoracic lymph node (012.10:0) |
|  |  | TB lymph node (017.20:0) |
|  |  | TB lymphadenitis (017.20:3) |
|  |  | TB periph lymph node by culture (017.24:0) |
|  |  | TB periph lymph node by micro exam (017.23:0) |
|  |  | TB periph lymph node- exam unknown (017.22:0) |
|  |  | TB peripheral lymph node by histo (017.25:0) |
|  |  | TB peripheral lymph node confirmed (017.26:0) |
|  |  | Varicella (052.9:0) |
|  |  | Varicella meningitis (052.7:1) |
|  |  | Varicella pneumonitis (052.1:0) |
|  |  | Viral disease (078.89:1) |
|  |  | Viral exanthem (057.9:0) |
|  |  | Viral hemorrhagic fever (065.9:1) |
|  |  | Viral infection (079.89:0) |
|  |  | Viral infection (079.99:0) |
|  |  | Viral rash (057.9:1) |
|  |  | Viremia (790.8:0) |
|  |  | Visceral herpes simplex (054.71:0) |
|  |  | Yellow fever (060.9:0) |
|  |  | Zika virus disease (066.3:8) |
|  |  | Zygomycosis or mucormycosis (117.7:0) |
